# Multi-omics in thoracic aortic aneurysm: the complex road to the simplification

**DOI:** 10.1186/s13578-023-01080-w

**Published:** 2023-07-20

**Authors:** Sara Rega, Floriana Farina, Silvia Bouhuis, Silvia de Donato, Mattia Chiesa, Paolo Poggio, Laura Cavallotti, Giorgia Bonalumi, Ilaria Giambuzzi, Giulio Pompilio, Gianluca L. Perrucci

**Affiliations:** 1 Unit of Vascular Biology and Regenerative Medicine, Centro Cardiologico Monzino IRCCS, Milan, Italy; 2 Unit for the Study of Aortic, Valvular and Coronary Pathologies, Centro Cardiologico Monzino IRCCS, Milan, Italy; 3 Institute for Cardiovascular Prevention (IPEK), Ludwig-Maximillians-Universität (LMU) München, Munich, Germany; 4 German Center for Cardiovascular Research (DZHK), Partner Site Munich Heart Alliance, Munich, Germany; 5 Bioinformatics and Artificial Intelligence Facility, Centro Cardiologico Monzino IRCCS, Milan, Italy; 6 Department of Electronics, Information and Biomedical Engineering, Politecnico Di Milano, Milan, Italy; 7 Department of Cardiovascular Surgery, Centro Cardiologico Monzino IRCCS, Milan, Italy; 8 Department of Clinical Sciences and Community Health, Università Degli Studi Di Milano, Milan, Italy; 9 Department of Biomedical, Surgical and Dental Sciences, Università Degli Studi Di Milano, Milan, Italy

**Keywords:** Thoracic aortic aneurysm, Epigenomics, Transcriptomics, Proteomics, Metabolomics

## Abstract

**Background:**

Thoracic aortic aneurysm (TAA) is a serious condition that affects the aorta, characterized by the dilation of its first segment. The causes of TAA (*e.g.*, age, hypertension, genetic syndromes) are heterogeneous and contribute to the weakening of the aortic wall. This complexity makes treating this life-threatening aortopathy challenging, as there are currently no etiological therapy available, and pharmacological strategies, aimed at avoiding surgical aortic replacement, are merely palliative. Recent studies on novel therapies for TAA have focused on identifying biological targets and etiological mechanisms of the disease by using advanced -omics techniques, including epigenomics, transcriptomics, proteomics, and metabolomics approaches.

**Methods:**

This review presents the latest findings from -omics approaches and underscores the importance of integrating multi-omics data to gain more comprehensive understanding of TAA.

**Results:**

Literature suggests that the alterations in TAA mediators frequently involve members of pro-fibrotic process (*i.e.*, TGF-β signaling pathways) or proteins associated with cell/extracellular structures (*e.g.,* aggrecans). Further analyses often reported the importance in TAA of processes as inflammation (PCR, CD3, leukotriene compounds), oxidative stress (chromatin OXPHOS, fatty acids), mitochondrial respiration and glycolysis/gluconeogenesis (*e.g.,* PPARs and *HIF1a*). Of note, more recent metabolomics studies added novel molecular markers to the list of TAA-specific detrimental mediators (proteoglycans).

**Conclusion:**

It is increasingly clear that integrating data from different -omics branches, along with clinical data, is essential as well as complicated both to reveal hidden relevant information and to address complex diseases such as TAA. Importantly, recent progresses in metabolomics highlighted novel potential and unprecedented marks in TAA diagnosis and therapy.

**Supplementary Information:**

The online version contains supplementary material available at 10.1186/s13578-023-01080-w.

## Background on thoracic aortic aneurysm

**Fig. 1:**
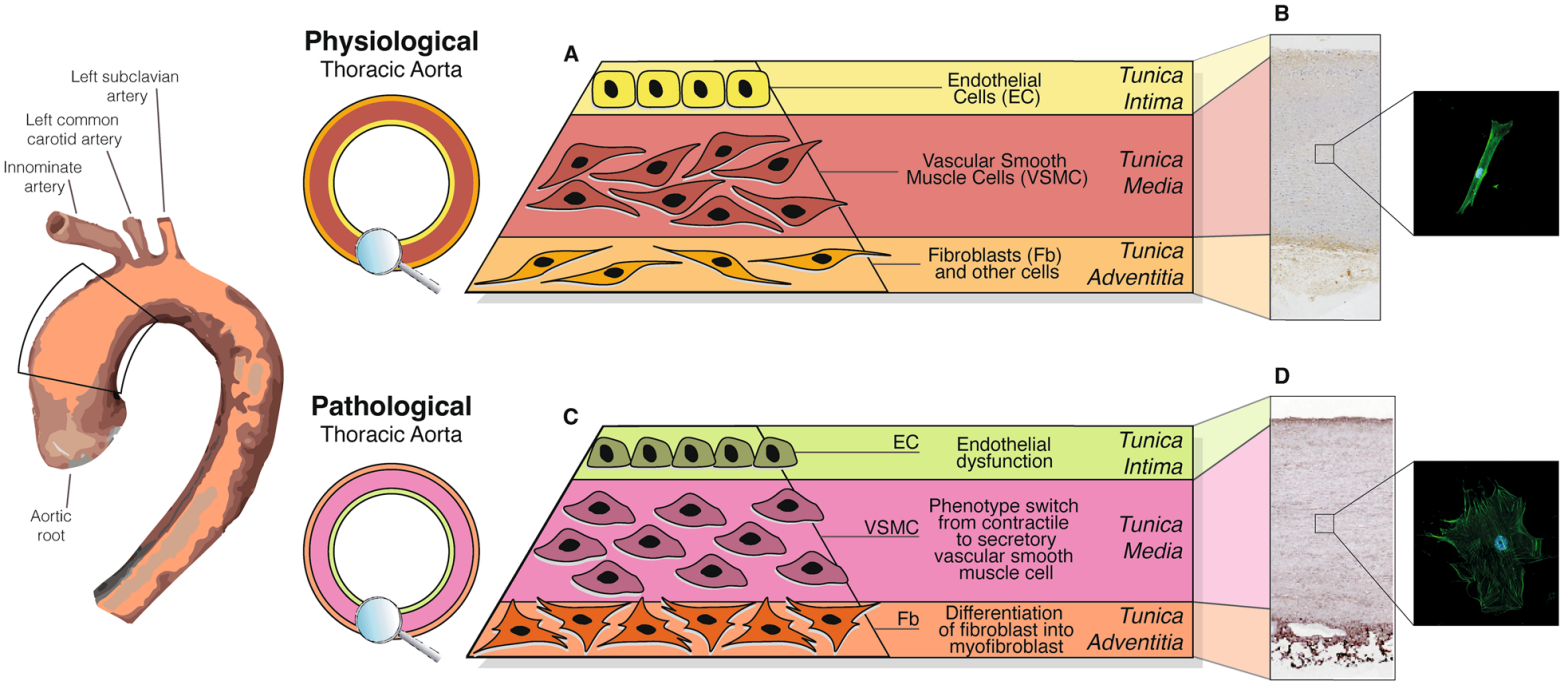
**–** Physiological and pathological features of ascending thoracic aorta. The ascending trait of thoracic aorta is the first region of the artery (left picture, black square), included between aortic root, which originates from the aortic valve, and the aortic arch at the level of brachiocephalic trunk (or innominate artery). **A**, **B** In physiological conditions, the wall of the ascending thoracic aortic trait is composed of three layers (*tunicae*) with distinct extracellular matrix composition, structure, and biomechanics. The inner layer, *tunica intima*, is defined by a cell lining of endothelium (EC), interfacing with the bloodstream. The medial layer, *tunica media*, is composed by the functional unit of the aortic wall, consisting in contractile vascular smooth muscle cells (VSMC), wrapped in elastic extracellular matrix. The outer layer, *tunica adventitia*, is an ECM-rich structure with adventitial fibroblasts (Fb). The physiological localization of collagen is mainly in *tunica adventitia* (**B**, left panel, brown staining) and VSMC are small and fusiform (**B**, right panel). **C**, **D** In pathological conditions, the three aortic layers may undergo detrimental alterations, reported in panel **C**, leading to aortic wall impairments, such as massive collagen deposition also in *tunica media* (**D**, left panel, brown staining) and phenotype switching of VSMC, from contractile to larger and secretive cells (**D**, right panel). Green signals in VSMC: phalloidin-FITC. Immunohistochemistry, VSMC isolation and immunofluorescence methods are reported in the “*Additional files*”

The aorta can be affected by either chronic or acute conditions, with aneurysms and atherosclerosis being the most common chronic conditions, while acute conditions include aortic dissection, aortic ulcer, and aortic hematomas [1]. Aneurysm is defined as the segmental dilation of a vessel, involving all the three wall layers, with at least 50% increase in the expected normal diameter, and the numerical values of the cut-off vary depending on the aortic region and age [2]. In some cases, aortic dilation can lead to lethal aortic dissection and/or vessel rupture [3]. The pathophysiology underlying aneurysms and aortic dissection is broad and not yet fully understood. Nevertheless, it is now well-established that any mechanisms that weakens the aortic *tunica media* and increases aortic wall stress can induce pathological dilation [3] (Fig. 1).

This review is in-depth focused on thoracic aortic aneurysms (TAA), with particular attention to those occurring in the ascending thoracic aortic trait, due to their higher frequency [4].

### TAA subtypes and epidemiology

Abdominal aortic aneurysms (AAA) are primarily associated with atherosclerosis [5], while TAA are linked to factors that modify the response of the thoracic aorta to biomechanical stimuli, such as ageing or genetic/biochemical alterations of aortic wall protein constituents. As a result, TAA can be further classified into three categories: (i) sporadic TAA (sTAA, *i.e.*, aneurysms based on de novo mutations or other comorbidities), (ii) familial TAA (*i.e.*, not associated with other pathological manifestations), and (iii) syndromic TAA (*i.e.*, aneurysms related with multiorgan disease manifestations, defined by syndromic contexts, as Marfan syndrome, MFS).

The first type, sTAA, arises from de novo gene mutations, inflammatory diseases, or traumatic conditions and [6] is primarily associated with risk factors, such as ageing [7–12] and hypertension [13–15]. Male sex is also a variable factor, due to testosterone activity which increases hydrogen peroxide generation and promotes the angiotensin II (AngII)–induced TAA development, by upregulation of AngII receptor type 1 (AT1R) expression [16, 17]. Therefore, hypertension exerts mechanical effects on the development of aortic disease. Additionally, rare copy number variants in gene regulating the adhesion and contractility of vascular smooth muscle cell (VSMC) have been identified in patients with non-syndromic sTAA [18–20]. In all these cases, the upregulation of common pathways, such as reactive oxygen species (ROS) production and stress signaling activation (*e.g.*, apoptosis, necrosis) can cause dysfunction and VSMC death, impairment of extracellular matrix (ECM) components, and aortic inflammation, all contributing to the TAA onset and progression [21].

Concerning the second TAA subtype, familial non-syndromic TAA, manifests a single pathological condition with a family inheritance pattern. The autosomal dominant transmission involves six different genetic *loci*, among which only three have been identified so far: *TGFBR2* (encoding transforming growth factor-β receptor type 2, TGFβR2), *ACTA2* (encoding α-smooth muscle actin, αSMA), and *MYH11* (encoding myosin heavy chain 11). The remaining loci are 5q13-14, 11q23.3-24, and 15q24-26 [6].

Lastly, the syndromic aneurysms are related to well-known genetic pathologies, such as MFS (caused by mutations in the FBN1 gene encoding for ECM protein fibrillin-1), Loeys-Dietz syndrome (LDS, related to mutations in TGFβR1 or TGFβR2), Ehlers-Danlos syndrome (EDS, caused by mutations in genes encoding COL3A1, TGFβR1 or TGFβR2), bicuspid aortic valve (BAV, the most common congenital heart malformation associated with loss-of-function mutations in *NOTCH1*), and Turner syndrome [6].

Obtaining an accurate estimate of the overall TAA prevalence in the general population is challenging, due to the clinical characteristics of these aortopathies. TAA is frequently clinically silent, and post-mortem findings widely vary among different studies. Nonetheless, a rise in its prevalence has been observed in the past decades [22], due to increased ability to detect the pathology as well as to relating ageing of the population [23]. The incidence of TAA is higher in patients older than 65 years of age, and, in this subset of patients, prevalence probably exceeds 4%. The estimated overall incidence across all age groups is about 6–10 cases *per* 100,000 person/year [22, 24]. The statistics might be more precise regarding TAA with genetic-determined causes, because of rarity of syndromes involved, but also in this case the data are far to be upgraded. TAA formation is, indeed, alone responsible of 95% of MFS fatal events (even in young age), and TAA, together with aortic dissection, constitutes the major cause of morbidity and mortality in MFS patients [25]. Recently, MFS demographic data report an estimated prevalence ranging from 1–3 in 10,000 live newborns, with no gender and ethnic tendency [26–28]. Approximately 75% of patients with the classic MFS phenotype have a family background of this disease, while the remaining 25% display de novo mutations [29]. The median age of MFS diagnosis is about 19 years, while TAA occurs at an average age of 34 years [30]; of note, about 50–80% of MFS patients will develop aortic dilation [31] and about 30% of them undergo aortic dissection.

### Current state of the art on therapy

Currently, there is no definitive treatment available for TAA formation and progression, and available pharmacological interventions, such as anti-hypertensive drugs, are considered only palliative measures [32]. Given the high incidence rate of TAA and the lack of a definitive therapeutic strategy, surgical replacement of dilated aorta is, to date, the only effective treatment. Importantly, the use of the surgical approaches generically underlies controversies: if thoracic aortic substitution is the only current effective tool in preventing lethal aortic dissection, on the other hand, it still remains an invasive and potentially harmful procedure and, moreover, there is still debate on the best timing for surgery [33].

Although the different pathophysiological subtypes of TAA, current treatment strategies primarily focus on hypertension management, lipid control, and smoking cessation [4]. Additionally, lifestyle modifications, such as limitation in intense physical activity, weightlifting restrictions, and pregnancy recommendations, are also advised, especially for syndromic patients (*i.e.*, serial follow-up echocardiograms, pregnancy counselling, prenatal test, planned caesarean delivery). Among medications to reduce blood pressure, the most administered to TAA patients are β-adrenergic receptor antagonists (β-blockers), AngII receptor blockers (ARB), AngII converting enzyme inhibitors (ACEI), and calcium channel blockers (CCB).β-blockers are still considered as first-line therapeutic option for syndromic (overall in MFS) and sporadic TAA [34], owing their ability to reduce the inotropic state of the heart, decrease the impact force of ejected blood on the aortic wall, and lower heart rate and blood pressure [4]. Early studies showed that the β-blocker propranolol decreased aortic dilation and mortality in MFS patients [35], however, large meta-analyses did not show the same beneficial effects [36, 37]. Nonetheless, β-blockers continue to be routinely prescribed to MFS-TAA patients to prevent the progression of aortic disease [38]. In case of intolerance to β-blockers, the anti-hypertensive ACEI, or CCB could alternatively be prescribed.

ARB are frequently administered in conjunction with β-blockers [39]. Losartan has been shown to prevent aneurysm progression in one MFS murine model. The effect was attributed to the reduction of TGF-β signaling and extracellular signal regulated kinase1/2 (ERK1/2) MAP kinase activation [40, 41].

Known to limit the production of AngII, ACEI reduce signaling acting through both AT1R and AT2R receptors. Clinical studies have shown that the ACEI Enalapril and Perindopril reduce aortic stiffness and aortic root diameter in MFS patients [42]. At last, CCB are to date prescribed to prevent aneurysm progression in MFS patients intolerant to β-blockers [43]. However, limited data are available on the effective efficacy and safety of their use for this condition.

### Novel potential targets based on in vitro and in vivo studies

All the aforementioned drugs are merely palliative treatments, since the only partial-resolutive therapeutic strategy for TAA is, to date, the surgical aortic replacement. During the last four decades, recent advances in surgical intervention have greatly improved the life expectancy of TAA patients [44]. The surgical approach for TAA depends on the exact localization of the aneurysm. It consists in replacing the diseased part with a vascular graft. In case of dilation of the tubular portion of the ascending aorta, the aortic root is preserved. In case of root aneurysms, the surgeon considers root and aortic valve replacement or solely root substitution [45]. Hence, the scientific community in the last years has been strongly focused on the research of other biological targets, not only to better understand the TAA characteristics and the underlying processes leading to onset and progression of dilation, but, more importantly, to identify potential novel therapeutics targets. Several studies have identified, indeed, novel biological items with this aim. These biological agents encompass molecules/mechanisms already involved in other pathophysiological processes, which pharmacological modulation may have positive impact in limiting TAA progression. However, their use may also carry the risk of adverse side effects in certain patients’ cohorts. While in vitro studies on TAA are mainly performed by using VSMC, several in vivo models are available, and are described in Additional file 1: Table S1.

In addition to their role as cholesterol-lowering mediators, 3-hydroxy-3-methylglutaryl coenzyme A reductase inhibitors (*i.e.*, statins) are also able in reducing inflammation and protecting the vascular wall [46, 47]. While long-term tolerability of statins can vary, they have shown effectiveness in preserving aortic structure and mitigating aortic root dilation in murine MFS models [48, 49]. Moreover, several clinical studies have indicated that statin use was consistently associated with improved long-term outcomes in patients with TAA [50].

Doxycycline, an antibiotic drug, is a potent matrix metalloproteinase (MMP) inhibitor [51, 52]. It has demonstrated to suppress the production and activity of MMP-2 and MMP-9, thereby reducing elastin degradation, and delaying AAA formation [53] or thoracic aortic rupture in MFS mice [54]. Moreover, doxycycline and Losartan have a synergistic effect in preventing the development of TAA in MFS murine models [55].

Several other compounds provided, in the last years, promising results both in vivo and in vitro within MFS-TAA context [56, 57]. Among worth of mention compounds, there are androgen inhibitors [58], folic acid [59], inhibitors of soluble guanylate cyclase [60], NAD [61], and apocynin [62], all displaying interesting in vivo outcomes by reducing TAA progression.

## -Omics application in TAA studies

Pharmacological therapies currently adopted in TAA context are tools limitedly able to contain the aneurysmal progression, thus delaying as long as possible the surgical aortic replacement to the patient. Only recently, research efforts have focused on the development of new TAA therapies, mainly aimed at identifying biological targets and understanding the underlying etiology of the disease, which still remains complex and not fully decrypted.

The various subtypes of thoracic aortopathy contribute to increase the intricacy of this pathological scenario as well as the complexity of the biological system, in which genetic factors, physical–mechanical stimuli and comorbidities with other pathologies may play a significative role.

To the end of rationalization, simplification, as well as targeting the research for etiological mechanisms of TAA, the adoption of different -omics disciplines seems to be mandatory.

These disciplines allow the dissection of the problem by several levels, in order to give an overview on genomic, epigenetic, gene transcription, proteomic, and metabolic alterations, typical of the specific pathology under study. This process determines, in an apparently counterintuitive manner, the generation of a multitude of data, which allows unmasking numerous pathological alterations in comparison with controls.

In details, epigenomics refers to the study of stable and heritable marks able to affect gene transcription, without modifying DNA sequence. Transcriptomics is the effective method to define differences among transcript profiling in certain tissues and/or cells. The transcript profile is also used to distinguish different cell types in tissues. Proteomics provide detailed information about the protein produced/secreted/released by a specific tissue region/cell type/subcellular fractions, of their activities and specific functions, as well as of their post-translational modifications. Lastly, metabolomics profiling measures the chemical processes involving metabolites, small molecule substrates, intermediates, and end-products of cell metabolism.

As mentioned, the multitude of available -omics and the dimension of generated data may further complicate already unclear pathological pictures, by tackling the problem through excessive layers. For this reason, several studies, to date, employ a combination of two or three of -omics approaches to achieve a more focused analysis. Recent advancement in technology, IT and computer power, such as big data handling, artificial intelligence and high-performance computing, facilitated the integration of multi-omics data. This has yielded in promising results in terms of determination of interaction networks or identification of unknown compromised mechanisms and patterns, potentially relevant for diagnosis and for targeted etiological therapy development. For all these reasons, the informatic step is largely considered increasingly necessary. Therefore, relying solely on a single -omics approach may no longer be sufficient to tackle complex problems.

In an intricate pathological scenario, such that offered by TAA, this novel approach may be helpful in the next years also in different TAA patients’ stratification and severe or fatal pathological outcomes prevention.

### Epigenomics studies

The epigenomic study is a branch of -omics focused on the evaluation of epigenetic modifications [63], including three main categories: (i) DNA methylation, (ii) histone modifications (Table 1), and finally (iii) post-transcriptional regulation of non-coding RNAs (ncRNAs) (Table 2). These changes exert a great impact on the recruitment of transcription factors and on the regulation of the transcriptional machinery, coordinating numerous biological processes, without altering DNA sequence [64]. Over the years, considerable evidence has demonstrated how lifestyle and environmental cues can trigger these alterations, resulting in numerous human diseases onset/progression. Additionally, epigenetic changes provide valuable insights into the development of new drugs, aimed at addressing the burden of these pathologies [65]. Next-generation sequencing studies allowed a more comprehensive and detailed view of gene regulatory pathways characterizing cellular identity. Furthermore, epigenetic cues have revealed further insights into the aberrant expression of many genes linked to the development and progression of many human pathologies, including cardiovascular diseases [66].

**Table 1:** Epigenomic studies focusing on DNA and histone modifications in aortic diseases

*DNA methylation*							
Aneurysm Type	Source		Target/modification		Pathways	Notes	Citations
	Species	Samples	Genes	Up/Down			
BAV-TAA, sTAA	Human	Aortic tissue	PTPN22	↓	T-cell receptor signalling	Global DNA hypermethylation	[68]
AAA in smokers and no smokers	Human	Mononuclear blood cells	CNN2	↓	Cytoskeletal organization and vascular development	Reduction in CpG methylation in AAA *vs* non-AAA	[69]
			SERPINB9	↑	Protection from granzyme B induced apoptosis	Increase in CpG methylation in AAA *vs* non-AAA	
AAA	Human	VSMC	SERPINB9	↓	Regulation of inflammation and apoptosis in VSMC	Increase in CpG methylation in AAA *vs* non-AAA	[70]
			SMYD2	↓	Pro-inflammatory cytokines production; T cell differentiation and apoptosis regulation	Reduction in CpG methylation in AAA *vs* non-AAA	
TAA or dissection	Murine	Aortic tissue samples from CBS^+/-^ mice	Dnmt1 and Dnmt3a	↑	Maintenance and de novo methylation		[72]
Intracranial aneurysm	Human	Peripheral whole blood	PNPLA6	↓	Neuronal differentiation	Increased DNA methylation	[73]
AAA and cerebral aneurysm	Human–Murine	Patients’ samples and VSMC from Uhrf1^−/−^ mice	UHRF1	↑	DNA methylation and histone post-translational modifications linkage	Increased global DNA methylation	[76]
*Histone post-translational modifications (HPMs)*							
TAA, BAV-TAA, and degenerative	Human	VSMC and fibroblast	H3K9/14ac and H3K4me	↑	SMAD2 overexpression in a TGF-ß1 independent manner	Sustained TGF-ß activity	[80]
TAA	Human	VSMC	H3K9/14ac	↑	SMAD2 hyperactivation	Histone acetylation, p53 recruitment, and acetylation	[81]

**Table 2:** Epigenomic studies focusing on non-coding RNA transcription modulation in aortic diseases

*Post-transcriptional regulation of short non-coding RNA*						
Aneurysm Type	Source		Target		Pathways	Citation
	Species	Samples	ncRNA	Up/Down		
sTAA	Human	Aortic tissue after balloon injury and VSMC	miR-21	↑	Apoptosis, neointimal iperplasia and VSMC phenotypic switch	[100, 101]
sTAA	Human	VSMC	miR-26a	↓	SMAD1/4 signalling	[96]
sTAA	Murine	VSMC	miR-26b-5p	↓	TGF-β/SMAD4 signalling	[102]
sTAA	Human	Aortic tissue	miR-29a	↓	ECM remodelling and degeneration	[88, 89, 96]
	Murine	Aortas from aged mice, aged AngII-infused mice and Fbln4^R/R^		↑		
MFS-TAA	Murine	FBN^C1039G/+^ mice aortic tissue	miR-29b	↑	ECM remodelling and degeneration, TGF-β signalling, apoptosis	[103–105]
sTAA, AAA	Rat	Atrium and cardiac fibroblasts	miR-30c-2	↓	TGF-β signalling	[87]
sTAA	Murine	Aortic tissue and EC	miR-126	↑	Angiogenesis	[85]
sTAA	Murine	Mouse and rat arteries and VSMC	miR-143/145	↓	VSMC differentiation, proliferation	[91, 92]
sTAA	Rat	VSMC and EC	miR-221/222	↑	VSMC differentiation, proliferation, migration and apoptosis (opposite in EC *vs* VSMC)	[97]
*Post-transcriptional regulation of long non-coding RNA*						
LDS-TAA	Human	Serum	AK056155	↑	TGF-β signalling	[114]
sTAA	Human	Aortic media and HAoSMC	GIVER	↑	Inflammation, oxidative stress, VSMC proliferation	[109]
sTAA	Human	Serum	HIF1A-AS1	↑	VSMC apoptosis	[106, 107]
sTAA	Human	HAoSMC	HOTAIR	↓	Cell proliferation	[115]
sTAA	Murine	ApoE^−/−^ mice aortas	(lincRNA)-p21	↓	Atherosclerotic plaques generation	[116]
sTAA	Human	Aortic tissue and VSMC	MALAT1	↑	VSMC apoptosis	[108]
sTAA	Human	Coronary artery VSMC	MYOSLID	↑	VSMC phenotype modulation, TGF-β signalling	[110, 111]
sTAA	Human–Murine	VSMC	XIST	↑	VSMC apoptosis	[112, 113]

At first, several studies were focused on DNA methylation, due to its important role in several biological processes and in the development of several human pathologies, such as cancer [67] and cardiovascular diseases [66], including the aneurysms.

Genome-wide DNA methylation studies allowed Shah and colleagues to identify several hypermethylated and downregulated genes such as protein tyrosine phosphatase, non-receptor type 22 (*PTPN22*). In addition, the group described the epigenetic regulation of the VSMC-specific *ACTA2* marker, without any reduced gene expression in patients with two different etiologies of TAA, namely patients with BAV-TAA and subjects with sTAA with tricuspid aortic valve (TAV), suggesting that DNA methylation could be crucial in the regulation of TAA development [68].

In addition, two independent studies demonstrated impaired methylation patterns in DNA, isolated from circulating mononuclear cells of AAA patients, for calponin 2 (*CNN2*) and serpin peptidase inhibitor clade B (ovalbumin) member 9 (*SERPINB9*) [69, 70]. Moreover, next generation sequencing revealed hypermethylation status of the *SMYD2* promoter in AAA patients-derived VSMC, highlighting a possible crucial role in the regulation of inflammation and adverse AAA outcome [70].

As previously mentioned, epigenetic mechanisms are influenced by environmental stimuli and lifestyle habits. In this context, a clear linkage was reported by Vats and colleagues in the strong correlation between hyperhomocysteinemia (HHcy), global DNA hypermethylation, and aortic dilation in a Swedish AAA cohort of patients [71]. Another study suggested that TAA patients might undergo matrix remodeling and homocysteine metabolism. This was further confirmed by the impairment in DNA methylation levels resulting from the actions of DNA methyltransferase (DNMT) 1 and DNMT3b in aortas from a HHcy mice (CBS^±^) [72].

Furthermore, increased methylation at phospholipase domain-containing protein 6 (*PNPLA6*), leading to its consequent reduction in mRNA expression, was found to affect intracranial aneurysm development [73].

Finally, in the regulation of DNA methylation and within the regulation of chromatin accessibility, the Ubiquitin-like with PHD and Ring Finger Domains 1 (UHRF1) protein, which play a crucial role in the regulation of DNA methylation and chromatin accessibility via the guidance of DNMT1 towards hemi-methylated DNA regions through a SET and RING-associated (SRA) domain, was found to be strongly upregulated in AAA and cerebral aneurysm patients [74]. In mice infused with porcine pancreatic elastase, UHRF1 expression was also significantly increased compared to controls. Genetic abrogation in vivo was sufficient to improve aortic wall homeostasis and radial arterial wall compliance in AngII-infused hyperlipidemic mice [75, 76].

A second mentioned mechanism of epigenetic modification involves alterations in chromatin scaffold proteins, such as histone post-translational modifications (HPTMs), which have a considerable impact on (i) the accessibility level of the transcriptional machinery on genes, (ii) chromatin physical properties, and (iii) the recruitment of histone modifiers or transcription factors. HPTMs encompass acetylation, methylation, phosphorylation, ubiquitylation, and SUMOylation. Among them, acetylation was the first to be discovered and is generally correlated with transcriptional activation. It mainly occurs on lysines (K), and some of the crucial marks are the acetylation of histone protein H3 at K9 and K27 (H3K9ac and H3K27ac) [77]. On the contrary, histone methylation varies according to the degree and site of methylation. Of note, tri-methylation of histone H3 on K9 and K27 (H3K9me3, H3K27me3) represents repression marks, whilst tri-methylation on K4 or di-methylation on K79 (H3K4me3, H3K79me2) are associated with transcriptional activation [78, 79].

Early evidence of potential epigenetic regulation in TAA development comes from the study of Gomez et al*.*, where an increase in H3K9/14ac with a concomitant augmentation in H3K4me3 at the *SMAD2* promoter was found in patient-derived VSMC. Taken together, these data reflect a transcriptional hyperactivation of *SMAD2*, leading to sustained activation of TGF-β pathway [80]. Additional studies, obtained from the same group, revealed that *SMAD2* hyperacetylation depends on the action of two histone acetyltransferases (HAT), *i.e.*, PCAF and p300. The authors further report a fine-tuning mechanism in which Myc-mediated transcriptional repression, present in healthy vessels, is replaced by p53, resulting in sustained transcriptional activity during TAA development [81]. Moreover, increased protease nexin-1 (PN-1) and plasminogen activator inhibitor-1 (PAI-1) expression in TAA patients was reported to be induced by SMAD2 binding and affect anti-proteolytic aortic SMC phenotype, thus leading to progressive aneurysmal dilation [82].

The role in aortic aneurysms of epigenetic alterations concerning non-coding transcripts (ncRNAs), of crucial importance in the regulation of gene transcription [83], has been investigated concerning all the ncRNA subtypes, including several short ncRNAs (*e.g.*, the micro RNA, miRNA) and long ncRNAs (lncRNA) [84].

Among miRNA, several of these molecules display common expression profiles in both TAA and AAA, such as the upregulation of miR-126 or the downregulation of miR-155 and miR-30c-2. The similarities of certain miRNA expression profiles in both TAA and AAA suggest their common involvement in pathological events leading to aortic dilation progression, such as the degenerative alterations of collagen and elastin matrix, or VSMC function impairment in aortic wall. More specifically, miRNA-126 is largely known to promote angiogenesis, but its direct influence in TAA or AAA is not yet clear [85]. On the contrary, miR-155 role in aneurysm context is better acknowledged, due to its link with atherosclerotic lesions and chronic inflammation [86]. In fact, miR-155 downregulation is consistent with the inflammatory nature of AAA, while in TAA, where medial degeneration occurs in the absence of inflammation, miR-155 deficiency may play an alternative role in TGF-β signaling regulation. Finally, the downregulation of miR-30c-2 has been shown to participate in fibrosis as a negative regulator of connective tissue growth factor (CTGF), a pro-fibrotic mediator promoting ECM component deposition [87].

Interestingly, Jones et al*.* [88] reported significant downregulations of several miRNA expression levels (*i.e.*, miR-1, -21, -29a, -133a, and -486) in human ascending TAA, and inverse correlations between their expression and the aortic diameter. Notably, miR-29a, was found to target MMP-2, suggesting that the upregulation of MMP-2 in TAA samples could be partially explained by the downregulation of miR-29a expression. Inversely, Boon et al*.* found a strong association between the augmented expression of miR-29 family members and the downregulation of ECM components, contributing to the aortic structure loss and, subsequently, to aneurysm development [89]. They also reported that these elevated expression levels were associated with a strong downregulation of numerous ECM components in the aortas of aged mice, as well as in two experimental models of aortic dilation (*i.e.*, AngII-infused mice and Fbln4^R/R^ model, Additional file 1: Table S1).

Apart from ECM degeneration, aneurysms also exhibit dysfunctional VSMC. It is to date well known that the VSMC switch their contractile phenotype into a synthetic state, and vice versa, in several pathological contexts. The former phenotype is characterized by high levels of contractile gene expression, with low rates of proliferation, migration, and ECM synthesis; on the contrary, the latter phenotype displays the opposite features, in terms of proliferation, migration, and protein synthesis. Several studies have nowadays identified miRNA expression profiles linked to VSMC differentiation or proliferation [90]. Contextually, Elia and colleagues provided evidence that not only the miR-143/145 cluster, but also miR-128 plays a crucial role in orchestrating VSMC development as contractile cells and their phenotypic switch, by targeting numerous transcription factors, including Kruppel‑like factor‑4 (KLF4), myocardin, and ETS Transcription Factor [91, 92]. In pathologic condition, this miRNA cluster is also able in regulating the VSMC phenotype switching [93]. Specifically, miR-143 inhibit cell proliferation by directly targeting K-RAS and ERK genes, which are associated with VSMC proliferation [94]. On the other hand, miR-145 promotes VSMCs differentiation and inhibits proliferation in sTAA [95]. Altogether, literature demonstrates that miR-145 can direct the smooth muscle fate and that this miRNA cluster can regulate the quiescent *versus* proliferative phenotype of VSMC [90]. Another important regulator of VSMCs phenotype is miR-26, as demonstrated by Leeper et al. [96], through a microarray-based study on human aortic SMC (HAoSMC) in vitro differentiation. Reduced levels of miR-26a were associated with decreased VSMCs proliferation and migration, as well as increased H_2_O_2_-induced apoptosis. Mechanistically, miR-26a targeted the expression of *SMAD1* and *SMAD4*, members of the TGF-β signaling cascade, thus affecting aneurysm development. After them, Liu et al*.* [97] isolated VSMC and endothelial cells (EC) from aortas of Sprague–Dawley male rats and observed the cellular downregulation of miR-221/222. Interestingly, these miRNA show a proactive role in cell proliferation and migration, as well as an anti-apoptotic effect on VSMC, but the completely opposite effects on EC. Moreover, miR-221/222 appear to directly target and downregulate p27 (*Kip1*), p57 (*Kip2*), and c-Kit, three proteins differentially expressed in VSMC and EC, all strongly involved in key processes of cell differentiation, proliferation, migration and apoptosis. Another highly expressed miRNA in VSMC is miR-21, known to be involved in the SMC regulation by its ability in targeting several cell fate-determination genes, such as *PTEN* [98], programmed cell death 4 (*PDCD4*) [97], and B cell lymphoma 2 (*BCL2*) [99]. In 2007, Ji et al*.* [100] found that miR-21 was one of the most upregulated miRNA in the vascular wall after balloon injury, and that its depletion significantly decreased neointima formation, suggesting a role for this miRNA as important regulator of neointimal hyperplasia. After that, in 2008, Davis et al*.* showed that miR-21 was also able in mediating the TGF-β- and bone morphogenetic protein (BMP)-induced contractile phenotype switch in human VSMC. At the same time, by its ability in downregulating PDCD4, miR-21 was acting as a negative feedback regulator of VSMC contractile genes. Moreover, the authors identified in this paper the transcription factor NF-kB as a crucial positive regulator of miR-21 expression in vascular cells. In fact, several molecules (*i.e.*, nicotine, IL-6, and AngII) were each able to induce miR-21 through NF-kB upregulation [101].

Recently, Changwu et al*.* reported that miR‑26b‑5p regulates the TGF-β/Smad4 signaling pathway in the modulation of hypoxia‑induced phenotypic switching in murine VSMC [102].

Merck and collaborators further revealed that miR-29b participates in early aneurysm development in MFS mice model Fbn1^C1039G/+^ (Additional file 1: Table S1) [103]. Moreover, miR-29b was found upregulated in both ascending aortic tissue and VSMC of these mice, and its inhibition prevented early aneurysm development, apoptosis of aortic wall cells, and ECM degradation [104]. In vitro studies confirmed the enhanced expression of miR-29b in VSMC isolated from Fbn1^C1039G/+^ mice, and further established that these cells are more prone to respond to excessive TGF-β signaling when compared to the cells of wild-type aortas. Further investigations on molecular mechanisms underpinning miR-29b expression recognized that the excessive TGF-β signaling upregulates miR-29b expression by decreasing the activation of NF-κB [105]. Therefore, it is possible to speculate that the increased miR-29b expression is the trigger for MMP modulation which, in turn, leads to ECM degradation and VSMC apoptosis.

Interestingly, apart from miRNA, the modulation of lncRNA has also emerged as a significant factor in understanding the molecular mechanisms underlying TAA pathology and identifying novel targets for TAA treatment. The initial study on differential lncRNA expression in this context highlighted the involvement of HIF1α-antisense RNA 1 (HIF1A-AS1) in TAA pathogenesis [106]. Indeed, the expression of HIF1A-AS1 was shown to be regulated by Brahma-related gene 1 (*BRG1*), whose levels are significantly higher in TAA when compared to healthy controls. Experiments on HIF1A-AS1 suppression resulted in reduced expression levels of caspase-3 and caspase-8, increased expression of BCL2, and attenuated palmitic acid (PA)-induced VSMC apoptosis. Furthermore, it has been reported that the HIF1A-AS1 expression was significantly increased in sera from TAA patients [107], suggesting its potential role as TAA biomarker. Also, VSMC apoptosis involved the regulation of several lncRNA, which resulted upregulated in TAA patients in aortic media specimens. The lncRNA-MALAT1, for example, interacts with *BRG1*, to regulate both VSMC apoptosis and function in TAA [108], but also with GIVER, who participates in inflammation and oxidative stress, and with its upstream activator LOXL1-AS [109]. Noteworthy, the LOXL1-AS/GIVER interaction is actively involved in the regulation of VSMC proliferation and apoptosis [110]. Recently, Zhao et al*.* [111] described a novel lncRNA, *i.e.* myocardin-induced smooth muscle lncRNA (MYOSLID), and its role in VSMC phenotype regulation. Indeed, MYOSLID, a direct transcriptional target of both MYOCD/serum response factor (SRF) axis and TGF-β/SMAD pathway, promotes VSMC differentiation. In details, the authors showed that MYOSLID depletion in VSMC was able to abrogate TGF-β1-induced SMAD2 phosphorylation, playing a role in TAA development by influencing the crosstalk between VSMC phenotype dysregulation and TGF-β signaling pathway activation. In recently performed in vivo experiments, Liang et al*.* [112] showed that elastin (Eln) inhibition protected rat VSMC from apoptosis. Also, miR-29b-3p was identified to bind to Eln, while X inactive specific transcript (Xist) could boost Eln expression through abrogation of miR-29b-3p. Furthermore, it has been observed that Eln overexpression counteracts the suppression of silenced Xist in rat VSMC apoptosis. Therefore, it could be summarized that *Xist*/miR-29b-3p/*Eln* axis appears to facilitate the apoptosis of mouse and rat aortic VSMC. Accordingly, the accelerating function of this axis was also verified in human TAA tissues (after dissection) and aortic VSMC [112, 113]. In MFS context, the co-expression network of Xist and miR-29b-3p identified Timp4 as a mRNA adjacent to Xist in Fbn1^C1039G/+^ aortas. XIST may participate in the mechanism of action behind MFS aneurysm by regulating the function of TIMP [103]. Still concerning the syndromic TAA context, Yu et al*.* [114] found that the lncRNA AK056155 was up-regulated in the patients’ serum with Loeys-Dietz syndrome (LDS), which correlated with an activation of the AKT/PI3K and TGF-β1 signaling. Finally, only the 5% of lncRNA were differentially expressed with significance in ascending aortic specimens derived from sTAA when compared with patients who undergone coronary artery bypass graft (CABG), often used as TAA controls. Among these, the lncRNA HOX transcript antisense intergenic RNA (HOTAIR) was a valuable marker candidate, since HOTAIR expression levels were significantly decreased in sTAA specimens. This data is of interest, because HOTAIR negatively correlates with aortic diameter. In vitro experiments confirmed that knockdown of HOTAIR induced both early and late apoptosis and reduced HAoSMC proliferation. Furthermore, both mRNA and protein expression levels of collagen types I and III were suppressed after HOTAIR knockdown [115]. Additionally, the long intergenic non-coding RNA (lincRNA)-p21 was shown to repress proliferation and induce apoptosis of VSMC isolated from atherosclerotic plaques of ApoE^−/−^ mice, stressing the significance of lncRNA even in sTAA [116].

### Transcriptomics studies

Despite the growing number of broad transcriptomics experiments comparing with healthy donors or other arterial tissues, there is a need of subtype-specific transcriptomics studies to obtain a deeper understanding of aneurysm pathology. However, such in-depth transcriptomics profiling remains limited and often incomplete, partly due to the choice of inadequate controls. Consequently, our knowledge regarding the transcription profiles of certain aneurysm subtypes, such as sTAA, remains poor, as summarized in Table 3.

**Table 3:** Analyses of transcript modulations in aortic diseases

Aneurysm Type	Source		Pathways		Notes	Citations
	Species	Samples	Target	Up/Down		
sTAA	Human	Aortic tissue (some with dissection) and left internal thoracic arteries	Inflammation-related pathways	*↑ CDH2*		[117]
				*↑ CYTL1*		
				*↑ SCG2*		
			Angiogenetic pathways	*↓ HOXA5*		
			Pro-fibrotic mediators	*↑ COL21A1*		
				*↑ HAPLN1*		
BAV-TAA, sTAA	Human	Non-dilated (nd) and dilated (d) aortic tissues from TAV and BAV patients	Cytoskeleton alterations	*↑ TAGLN2* in TAV (d *vs* nd)	Multivariate Data Analysis of Protein Expression Data	[118]
				*↑ ACTN1-4* in d *vs* nd		
				*↑ MFAP4* in d *vs* nd		
				*↓ MYL6* in d *vs* nd		
BAV-TAA, sTAA	Human	Non-dilated and dilated aortic tissue	Valvular calcification	*↓ NOTCH1* in BAVc and TAV	BAVc = calcific BAVr = with redundant leaflets	[119]
			Proteolytic/elastolytic activity	*↓ ADAMTS9* (in BAVr)		
				*↓ ACAN* in BAVr		
				*↑ MMP-9* in BAVc and TAV		
MFS-TAA	Human–Murine	Skin fibroblasts	TGF-β activity; ECM structure/turnover	*↓ VDR*		[123]
				*↑ TSC2*		
				*↑ LMO7*		
MFS-TAA	Murine	Aortic root, aortic tissue and VSMC from MFS Fbn1^C1041G/+^ and ApoE^−/−^ mice	ECM modulation and collagen synthesis; cell adhesion and proliferation	*↑ Loxl1*		[125]
				*↑ Ctgf*		
				*↑ Comp*		
				*↑ Thbs1*		
				*↑ Klf4*		
				*↑ Serpin1*		
MFS-TAA	Human–Murine	Aortic tissue from Fbn1^mgR/mgR^	VSMC phenotype switch and differentiation	*↓ Klf4* in young mice, time-dependent increase		[127, 128]
		MFS-hiPSCs differentiated into lateral mesoderm (LM), paraxial mesoderm (PM) and NC cells		*↑ KLF4*		
MFS-TAA	Human–Murine	Aortic root and VSMC from MFS-TAA and aortic tissues from Fbn1^mgR/mgR^ mice	VSMC contractility	*↑ ACTA1*	System pharmacology–based integration of human and mouse data for drug repurposing	[130, 148]
			ECM components	*↑ ACAN*		
MFS-TAA	Human–Murine	Patients’ and Fbn1^C1039G/+^ aortic tissues	Mitochondrial function	*↓ Tfam*		[61]
				*↓ Ppara*		
				*↓ Pparg*		
			Glycolytic metabolism	*↑ Hif1a*		
				*↑ Myc*		
sTAA	Human	Aortic tissue and VSMC	Pro-apoptotic and anti-inflammatory pathways	*↓ ERG*		[131]
			Mitochondrial function	↑ Chromatin OXPHOS		
sTAA	Human	Datasets	Inflammation-related pathways	*↑ CD3*	Bulk Transcriptome and Single-Cell RNA Sequencing Data integration	[132]
				*↑ ITGAM*		
			Pro-fibrotic pathway	*↑* TGF- β pathway		
MFS-TAA, sTAA	Human–Murine	Aortic tissues from 24-week old Fbn1^C1039G/+^ and ApoE^−/−^ mice	VSMC phenotype switch and differentiation	*↑ Fn1*	DEG analysis and Single-Cell RNA Sequencing Data integration	[125]
				*↑ Mgp*		
				*↑ Nupr1*		
				*↑ Eln*		
				*↑ Col1a1*		
				*↑ Ctgf*		
				*↑ Serpine1*		
		25-year-old male MFS patient aortic root		*↑ COL1A1*		
				*↑ CTGF*		
				*↑ SERPINE1*		

In 2017, Sulkava et al*.* discovered upregulation of several chemotactic genes in sTAA compared to healthy samples. Among these genes, there are cadherin 2 (*CDH2*), an indicator of mesenchymal stem cells [117], cytokine-like 1 (*CYTL1*), and secretogranin 2 (*SCG2*), markers of trans-endothelial migration of leukocytes. The upregulation of these genes leads to the enhanced vascular permeability and loss of integrity in the aortic wall, rendering the aorta susceptible to aneurysm or even progression to dissection. On the contrary, the downregulation of some genes, *e.g.* Homeobox A5 (*HOXA5*), has been proven to be important in the angiogenetic context [117].

The only available transcriptomics data on aneurysms caused by genetic mutations mainly concern patients affected by BAV and MFS patients. Several studies tried to characterize the transcriptome differences between BAV *vs* TAV by using RNA-seq technique. In 2013, Kjellqvist and colleagues highlighted interesting transcriptomic differences between dilated and non-dilated aortic regions of patients with sTAA and BAV-TAA. They observed differential gene expression related to cell structure and cytoskeleton mediators. Specifically, the authors observed in the dilated aortic region higher expression of *ACTN1*, *ACTN4* as well as *MFAP4* compared to non-dilated segments in both patient cohorts, while MYL6 gene, associated with the contractile phenotype of VSMCs, showed downregulation in the dilated zone [118]. Other authors have focused their studies on pro-calcific mediators to underline differences between sTAA and BAV-TAA. Among them, Padang et al*.* described differential gene expression profiles among BAV patients, distinguishing those with a calcification-predominant disease (BAVc, more similar to the pathologic phenotype of patients with TAV) and those with primarily redundant leaflet degeneration (BAVr). Both BAVc and TAV show notable downregulation of the NOTCH1 signaling pathway, underpinning a common terminal pathway in genes regulating valvular calcification. Furthermore, downregulation of *ADAMTS9* and aggrecans (ACAN) was observed in the BAVr compared with the TAV, while significant increase in MMP-9 activity was found in heavily calcified leaflets of both TAV and BAVc. Taken together, these data explain the enhanced proteolytic/elastolytic activity in ECM of BAV patients [119].

Furthermore, Di Vito et al*.* provided evidence on calcification event similarities between BAV and TAV. In both these pathological contexts, the aortic side of the valve leaflets displays active biological processes, involving inflammation, oxidation, angiogenesis, ECM remodeling/fibrosis and even atherosclerosis-like ectopic calcification with bone-like mineralization [120].

Although VSMC phenotype switching toward a synthetic state and the subsequent ECM modulation is a core concept in MFS-TAA formation [32, 121, 122], a comprehensive in vivo transcriptomics to asses this process is lacking. In 2007, Yao et al*.* observed that the transcription of genes encoding vitamin D receptor (*VDR*), a negative regulator of TGF-β transcriptional activation [123], and tuberous sclerosis complex 2 (*TSC2*), a potent activator of TGF-β, are significantly altered in MFS skin fibroblasts (Fb) when compared to healthy control cells. In details, due to their opposite functions, *VDR* levels were decreased, while *TSC2* levels were increased in MFS skin cells *vs* control. Another gene, *LMO7* (a regulating protein of cell adhesion) is induced by TGF-β and is significantly elevated in MFS-Fb. The behavior of all these three genes, together with other genes involved in ECM structure and turnover (*e.g.*, *ADAM12*, *MMP1*, *TIMP3*, *COL3A1*, *COL1A2*, *PLOD2*), is consistent with the enhanced TGF-β activity and the authors suggested their role in contributing to TAA development in MFS patients [124]. Accordingly, Pedroza et al*.* [125] have recently observed higher expression levels of TGF-β1 ligand, lysyl oxidase-like 1 (*Loxl1*), and fibrosis-related genes, such as *Ctgf* (connective tissue growth factor), *Comp* (cartilage oligomeric matrix protein), and *Thbs1* (thrombospondin-1) in a MFS murine model (Fbn1^C1041G/+^). These findings supported the massive collagen deposition as a consequence of pro-fibrotic phenotype [126], as well as several alterations in genes related to elastic fiber synthesis (*Eln*, *Fbln2*, *Fbln5*, *Fbn1*), laminins (*Lamc3*, *Lama2*) and fibronectin (*Fn1*) [125]. Bioinformatic tools enabled the prediction of upstream transcription factors that may promote in vitro VSMC modulation, including *Klf4* and *Sp1*, both potentiating PDGF-mediated VSMC phenotype change [127]. In details, *KLF4* overexpression has been identified in aortic tissue specimens of MFS murine models and in iPSC-derived VSMC [128]. Conversely, reduced levels of *Klf4* have been found together with deleterious overexpression of contractile markers in young MFS Fbn1^mgR/mgR^ mice [129]. Despite upstream drivers of Klf4 gene expression in MFS are still unknown, these reports suggested the dynamic temporal regulation of both *Klf4* expression and VSMC phenotype in MFS. The expression impairment of genes related to VSMC contractility in Fbn1^mgR/mgR^ mice has been also observed in a recent study of Hansen et al*.* [130], who observed and tested the potential efficacy of baclofen (*i.e.*, a GABA_B_ receptor agonist) in reducing TAA dilation. The therapeutic potential of this compound was the result of an elegant strategy, involving the integration of transcriptomics data with computational drug prediction analysis on thoracic aortic samples obtained both from MFS mice and patients [130]. In a recent work of 2021, Ollel et al*.* found that aortas of Fbn1^C1039G/+^ mice and human MFS patients display low *TFAM* expression, below normal mitochondrial DNA (mtDNA) levels, together with a mitochondrial respiration decline. The reduction of oxidative phosphorylation was caused by an extracellular tuning of mitochondrial respiration and it was compensated by increased glycolytic metabolism [61]. Since this process contributes to the first steps of aneurysm onset, it could be a suitable parameter for monitoring MFS patients during time.

Recent single-cell RNA sequencing (scRNA-seq), an effective tool enabling the transcriptome analysis of thousands of single cells, provides information about heterogeneity and cell-specific dynamics, useful in TAA pathological context both to decrypt the cell composition of TAA and also to compare the gene expression in cells from healthy *vs* aneurysmal aortas. For instance, in 2020, Li and colleagues [131] performed a scRNA-seq analysis on ascending aortic tissues from eight patients with sTAA and three control subjects. They identified eleven major cell types in the ascending aortic wall, exhibiting a distinct gene expression profile. Among the main cell types, two different clusters of VSMC in addition to Fb, mesenchymal stem cells, EC, monocytes/macrophages/dendritic cells, T lymphocytes, natural killer cells, mast cells, B lymphocytes and plasma cells are acknowledged. The study revealed a loss of non-immune cells (*i.e.*, VSMC, EC, Fb, and mesenchymal stem cells) and an increase in the number of immune cells in the TAA wall. Integrating the scRNA-seq data with GWAS, the authors identified eleven differentially expressed genes associated with aneurysms including *TWIST1*, *ADAM15*, *ERG*, *UBE2Q1*, *TPM3*, *ATP8B2*, *C1orf43*, *RSAD2*, *DNM2*, *KANK2*, and *HAX1*. Notably, decreased expression of the transcription factor *ERG*, and mitochondrial dysfunction were observed in various cell types within TAA tissues. *ERG* downregulation in VSMCs, inflammatory cells, Fb, and EC suggests its potential role as positive regulator of anti-apoptotic genes and negative regulator of pro-inflammatory genes. Despite mitochondrial gene expression being reduced in several cell types, chromatin oxidative phosphorylation (OXPHOS) gene expression was paradoxically increased, potentially as a compensatory mechanism to maintain critical OXPHOS functions. The study also confirmed the previously mentioned observation of increased glycolysis-related gene expression in stressed VSMCs within TAA tissues [131]. In 2022, Wang et al*.* [132], taking into advantages of available datasets of differentially expressed genes, observed that sTAA samples were enriched in several immune-related pathways (*e.g.*, TGF-β signaling pathway, leukocyte trans-endothelial migration) in comparison with normal thoracic aorta. This result, consistent with the previous work, confirmed the correlation between immune responses and sTAA progression. In details, the scRNA-seq performed in this work displayed in sTAA a lower number of VSMC and, on the contrary, a higher number of immune cell populations, especially macrophages and T cells. In addition, they found that the inflammatory response induced by macrophages facilitates the initiation and progression of sTAA [132]. The previously mentioned paper of Pedroza et al*.* [125] showed a scRNA-seq analysis performed on 24-weeks old Fbn1^C1041G/+^ mice and on littermate healthy controls, in order to describe cell-specific transcriptomics changes associated with advanced MFS aortic aneurysms. The differential expression gene analysis determined a peculiar cluster of modulated VSMC, defined modSMC (*i.e.*, VSMC with transcriptome modulated toward a Fb-like state). These cells were only identified in the aortic aneurysm tissue of adult Fbn1^C1041G/+^ mice. The specific features of modSMC included the expression of SMC markers (*Acta2*, *Myl9*, *Myh11*, *Tpm2*), together with stronger expression levels of fibronectin 1 (*Fn1*), matrix gla protein (*Mgp*), nuclear protein 1 (*Nupr1*) and the previously mentioned *Eln*. The comparison of Fbn1^C1041G/+^ thoracic aorta with atherosclerotic aorta of the ApoE^−/−^ mice revealed similar patterns of SMC modulation, but further led to the identification of an MFS-specific gene signature, which included also plasminogen-activator inhibitor 1 (*Serpine1*) and *Klf4*. To better compare the findings obtained from the analysis of Fbn1^C1041G/+^ mouse, the authors further performed a scRNA-seq study on aortic root tissue obtained from a 25-year-old male MFS patient. Both mouse and human scRNA-seq data demonstrated a significantly increase in *Col1a1* in the modulated SMC subset. Furthermore, enhanced expression of *Ctgf* and *Serpine1*, both markers of active TGF-β signaling, was associated with modSMC identity. Expression of *Tgfb1* ligand was also significantly enriched in modSMC within both datasets [125]. Noteworthy, an interesting detailed review on scRNA-seq studies in TAA pathological context has been recently published by Mizrak and colleagues [133].

### Proteomics studies

**Fig. 2:**
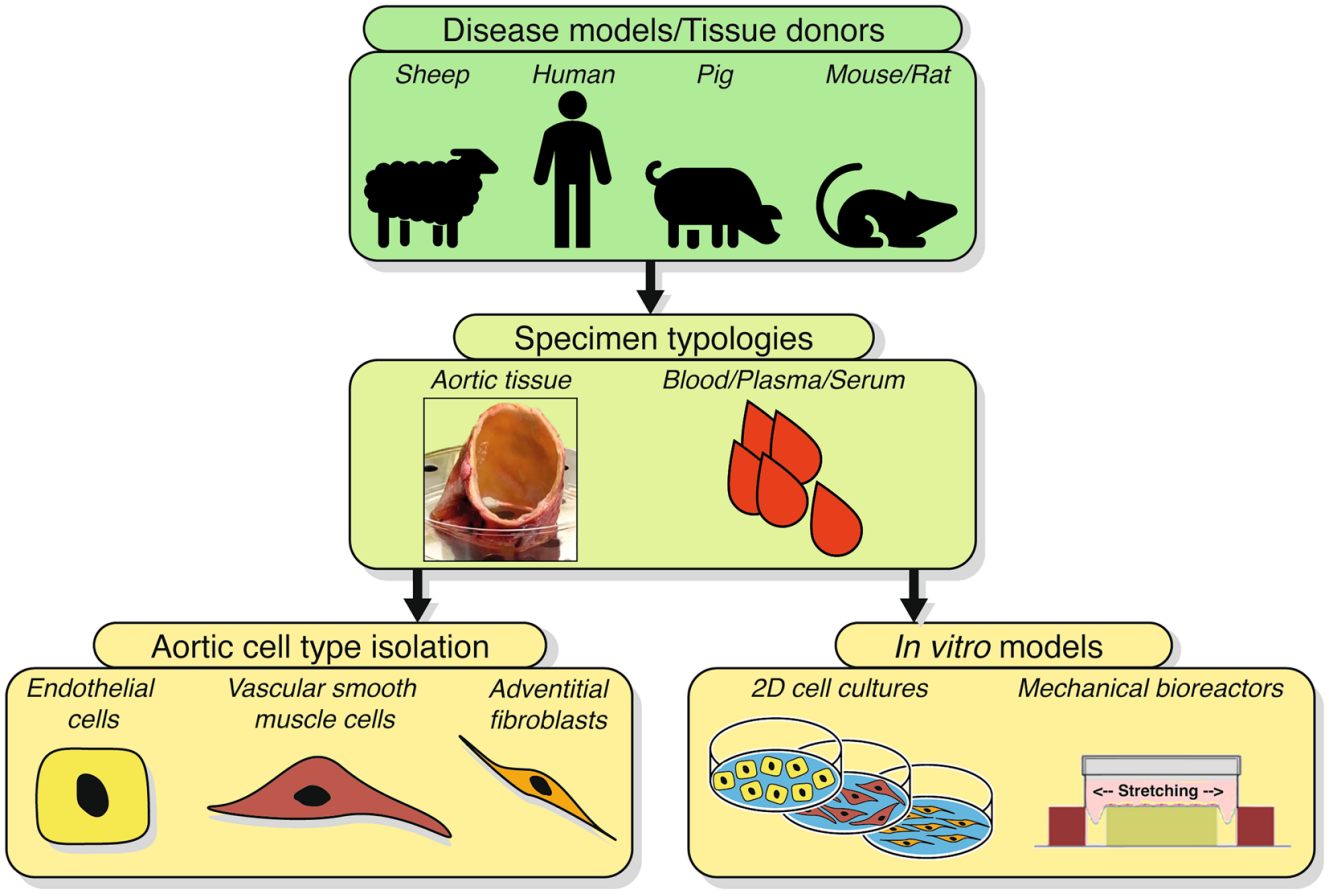
**–** Workflow for thoracic aortic disease study. To conduct a proper multi-omics study of thoracic aortic disease, a clear and defined workflow must be followed, ranging from the proper selection of the disease model to the correct analysis of the resulting -omics data. Of crucial impact is the collection of thoracic aortic tissues or biofluids such as whole blood, plasma, or serum from both human donors as well as from a number of available well-defined animal models of aortopathies (Additional file 1: Table S1). Attention should be paid to the enrolment of the proper healthy controls and to correctly dissect different pathological conditions (*e.g.*, sporadic and syndromic aneurysms). Depending on experimental aims/contexts, these samples can be used for aortic cell isolation and culture in standard 2D in vitro supports as well as in specific bioreactors

As for transcriptomics studies, one of the major issues encountered by reviewing proteomics works concerns which type of specimens (in terms of source and organism) and, overall, controls have been adopted for the comparisons with the pathological samples (Fig. 2).

The first study, in chronological order, focused on distinguish differentially expressed molecules between human aortic samples obtained from TAA patients and healthy controls by using proteomics approach is the work of Black et al*.* in 2013 [134]. The paper was aimed to unveil the proteomic differences among aneurysmal aorta and controls, but also to find out a potential circulating pathological signature, in terms of mRNA.

Concerning the proteomics analysis, TAA samples were obtained from a mixed population, including patients with BAV and TAV of both genders, whereas as controls aortic tissue from healthy donors have been used. Interestingly, the work differentiates the results by stratifying the population by means of aneurysm size (*i.e.*, small-, medium-, and large-sized TAA). The results suggest that calponin-1, plastin-3, and peptidyl-prolyl *cis–trans* isomerase were commonly up-regulated in all TAA size groups. Interestingly, in small and medium-sized TAA (*i.e.*, from 4.0 to 5.5 cm of diameter) the up-regulated pathways were those related to cytoskeletal protein binding, actin binding, muscle organ development and glycolysis/gluconeogenesis pathways, while in the large TAA (*i.e.*, > 5.5 cm) the statistically higher expressed pathways were those related to acute inflammatory response, structural molecule activity, ECM and acute-phase response. Regarding the latter aspect, Kim and colleagues [135] have recently observed the C-reactive protein (CRP) levels in aortic and serum samples of AAA patients, in order to determine the association between the degree of monomeric CRP (mCRP) deposited in the damaged aortic walls and the proteomic changes of tissue. Here, AAA samples have been compared with specimens obtained from patients with ascending thoracic aortic dissection, used as negative controls for absence of atherosclerosis. In this study, serum CRP levels of controls were significantly higher when compared with AAA. However, counterintuitively with results of Black, TAA showed only a small deposition of total CRP and mCRP in the aortic tissue. A possible explanation could be linked to the effector role of CRP in acute-phase response. The interesting result obtained by Black and colleagues was further supported by several metabolomics studies, discussed hereafter in paragraph 2.4. In details, the specific pathway involving phenylalanine, tyrosine, and tryptophan biosynthesis was overall commonly up-regulated in TAA [134]. The analysis performed by Chiarini et al*.* in 2018 further confirmed in sTAA, when compared with healthy aortic specimens, the alterations in proteins involved in cytoskeleton structure, enzyme and cell signaling [136]. These data were justified by the authors by an intensification of Jagged1/NOTCH1 signaling pathway in aortic SMC.

The enriched canonical pathways identified by Ingenuity Pathway Analysis (IPA) performed by Abudupataer and colleagues, in 2021 [137], showed a significant enrichment in TAA tissues of acute phase response signaling, mitochondrial dysfunction (*e.g.*, EIF2 and sirtuin signaling pathways) and oxidative phosphorylation pathways. Noteworthy, this work was focused on the comparison between BAV-TAA only and healthy aortic samples.

Recently, an interesting work of Tyrrell et al*.* [138] demonstrated a more significant differential expression of proteins by comparing healthy aged and young aortas in respect with aged and young TAA, almost all with BAV. The authors found that aging processes lead to an enforcement of metabolic pathways and alterations in the proteome, both in the comparisons between healthy and diseased aortas, but also between aged and young healthy aortas. Noteworthy, young samples generally show an enrichment in immunologic processes in comparison with healthy and aneurysmal aortas. However, the authors acknowledged the need for further mechanistic studies to determine the precise indication of role of any identified proteins in TAA pathogenesis.

The importance of choosing a different typology of control may affect the scientific findings, somehow leading to discordant results with other studies. The results of Kjellqvist et al*.* [118], published before Tyrrell and colleagues, suggested that aortic dilation in patients with a TAV mainly involves inflammatory processes, while patients with BAV develop aortic aneurysms as consequence of repair impairments. Of note, this study analyzed thoracic aortic samples obtained from patients with TAA by comparing patients both with TAV and BAV, but normalizing the results on aortic samples of patients who did not undergo aortic replacement, but only aortic valve repairment/substitution.

A comprehensive proteomics study on aortic tissue samples has been performed in 2015 by Matsumoto and collaborators [139]. Here, the authors provided a large proteome analysis by the comparison between TAA/AAA with respective adjacent non-dilated aortic portions. Concerning only TAA results, the dilated zone shows overexpressed levels of ECM proteins (*e.g.*, fibrinogen, tenascin, thrombospondin-1, decorin, vitronectin) in comparison with non-dilated region, whereas downregulated proteins are cytoskeletal proteins (*e.g.*, myosin regulatory light polypeptide 9, destrin, filamin-binding LIM protein 1, transgelin, vimentin, myosin light polypeptide 6). A further interesting, but counterintuitive, result of this study concerns the upregulation of α-2-macroglobulin, suggesting a potential role of this molecule as physiological “buffer” against TAA. In fact, the authors found that the α-2-macroglobulin expression was increased in TAA regions when compared with non-dilated tissue, potentially justified by its known function as active proteases inhibitor. Furthermore, the MMP activity resulted particularly high in thoracic and abdominal aneurysms. Specifically, in this case a further comparison of these samples with healthy control specimens may be useful to confirm the results and explain the discovery about the role of α-2-macroglobulin in TAA context.

Frequently, studies on TAA adopted another source of “controls”, such as aortic samples obtained by patients underwent coronary artery bypass grafting (CABG). Both the studies of Serhatli and colleagues in 2014 and Zhang et al*.* in 2015 showed results on differential expression of proteins by comparing TAA with CABG samples, which was used as controls [140, 141]. The difference among these two mentioned studies is that Serhatli extracted the total proteins by microdissecting the *tunica media* from frozen tissue sections, whereas Zhang from total aortic tissue, freshly obtained from surgery room. In the first study, which actually analyzed only the components of the *tunica media*, downregulated proteins in TAA group *versus* CABG were mainly associated to cytoskeletal, ECM components, protein involved in ECM remodeling, cellular organization, maintenance, morphology, movement and cell-to-cell signaling and interaction [140]. The study of Zhang displays that upregulated proteins in TAA *versus* CABG were components of ECM and proteins related to cytoskeletal structures, cell attachment, adhesion, migration and organization, cell-to-cell and cell-to ECM signaling, whereas downregulated proteins were molecules involved in different pathways (*i.e.*, complement activation, platelet aggregation, LDL trafficking) and function related to ECM and cytoskeleton organization [141].

A paper of 2017 authored by Rocchiccioli et al*.* [142] is based on the study of secreted factors from tissue, which determine a boundary between studies performed on tissue and those focusing on circulating factors afterward discussed. Here, Rocchiccioli and colleagues cultured patient-derived aortic specimens (BAV *vs* TAV) in a serum-free medium and then analyzed the released factors by proteomic and real-time PCR. Importantly, among TAA patients with TAV there were no syndromic patients. The results of the work just confirmed the crucial role played by TGF-β signaling pathway in BAV-TAA. More specifically, among protein modulated in BAV aneurysms, 21 out of 38 were involved in TGF-β activation, such as LTBP4, which was strongly linked to TGF-β storage in ECM and under-expressed in BAV samples.

Regarding one of the most studied syndromic TAA, such as MFS-derived TAA, there are two studies worth of mention. In 2019, the study of Yin et al*.* investigated a large subgroup of ECM constituents, by analyzing the differential glycoproteomics between MFS-TAA and non-syndromic sTAA [143]. Here, the most significant differences between syndromic and sporadic ECM were observed for microfibril-associated glycoprotein 4 (MFAP4), as well as for the two large aggregating proteoglycans, as aggrecan (PGCA) and versican (CSPG2).

The second important study analyzing MFS-TAA tissues is the recently published work of Verhagen et al*.*, which also represent an effective and representative example of -omics integration study [144]. The authors here, by comparing proteomics and transcriptomics data, indicated that all the mediators within mitochondrial respiration network were underrepresented in MFS *versus* controls, thus the alterations between syndromic and non-syndromic TAA originated from mitochondrial dysfunction, especially in MFS patients with *FBN1* haploinsufficiency. The alterations in the expression levels of mitochondrial constituents have been reported also by other authors in sTAA contexts [145].

In 2013, Satoh et al*.* [146], in order to identify potential biomarkers for aortic aneurysms, analyzed circulating proteins in the serum of patients undergoing AAA and TAA substitution, comparing the proteomic differences before and after surgery. The results of this study lead to the conclusion determining kallistatin and α-2-macroglobulin as potential circulating markers in the serum of both AAA and TAA, or only in TAA patients, respectively. Unfortunately, the paper did not in-depth indicate the TAA origins, thus it did not discriminate patients with genetic disorders and subjects with sporadic or familiar TAA. The previously mentioned study of 2015 performed by Matsumoto et al*.* [139] partially confirmed the Satoh results, since they reported a consistent alteration of serum levels of only α-2-macroglobulin, but not of kallistatin.

An interesting comparative analysis, published in 2021 by Ma et al*.*, yielded a total of 193 differentially expressed proteins between BAV patients with TAA in respect to controls [147]. More specifically, the authors performed (i) a disease enrichment analysis (revealing alterations in mediators typical of vascular diseases and abdominal aortic aneurysm), (ii) an integrated enrichment analysis using Metascape (showing a network of enriched terms, *e.g.*, regulated exocytosis, platelet degranulation, extracellular structure organization) [148], and (iii) a canonical pathway enrichment analysis using Ingenuity Pathway Analysis (IPA) software (where differentially expressed proteins were related to epithelial adherent junction, RhoA, integrin, and actin cytoskeleton signaling pathways). Among the upstream regulators, Ma and colleagues identified ADAM-10 (a known regulator of NOTCH signaling pathway), MMP-12, TGF-β1, and GATA4, but the conclusions of the work suggest the potential for plasma proteins, such as NOTCH3 and ADAM-10, as predictors of aortic dilation [147].

The recently published paper of König et al*.* [149] was aimed to determine a potential biomarker for the progression of TAA in aortic dissection. So, the authors compared plasma samples obtained from patients with TAA and from patients who occurred an aortic dissection event. Importantly, the patients’ cohort of this study acknowledged not only subjects with sTAA and TAV, but also syndromic patients (*i.e.*, MFS) and BAV. Although the strong upregulation of aggrecan (*ACAN*) transcript in the aortic tissues of TAA patients, previously observed also by Cikach [150] and later discussed, they have identified ACAN plasma levels as a reliable biomarker to detect the presence only of the thoracic aortic dissection in a very sensitive manner.

Literature do not frequently offer proteomics studies on VSMC, potentially due to the difficulties of working with primary human VSMC. Nonetheless, for what it concerns studies on specific TAA types and more specifically those with genetic determinants, an elegant study has been recently published by Iosef et al*.* in 2020 [151], by performing a proteomic characterization on iPSC-derived MFS-VSMC. The authors produce in this work MFS patients’-derived iPSC, then they differentiate VSMC from two different embryonic derivations, such as the lateral mesoderm and the neural crest. Mannose receptor C type 2, transgelin and nestin are examples of downregulated proteins in MFS-VSMC when compared with healthy controls, while the upregulated molecules resulted integrins, MMP-2, type 1 collagen α1, and fibronectin 1. All these studies have been outlined in Table 4.

**Table 4:** Proteomic studies in aortic diseases

Aneurysm Type	Source		Pathways		Notes	Citations
	Species	Samples	Target	Up/Down		
BAV-TAA, sTAA	Human	Aortic tissue and whole blood	Cytoskeletal binding, organ development, glycolysis/gluconeogenesis pathways (small-, medium-sized TAA) and acute inflammatory response and ECM remodelling (large-sized TAA)	↑ Calponin-1	Small-, medium- and large-sized TAA stratification	[133]
				↑ Plastin-3		
				↑ Peptidyl-prolyl cis–trans isomerase		
AAA, AAD	Human	Aortic tissue and serum	Atherosclerosis pathways and tissue remodelling	↑ C-reactive protein (in serum)	AAD used as controls	[134, 135]
				↓ C-reactive protein (in AAD tissue)		
sTAA	Human	Aortic tissue and VSMC	Cytoskeleton structure and signalling pathways	↑ Jagged1/NOTCH1 signalling		[136]
BAV-TAA	Human	Aortic tissue and HAoSMC	Acute-phase response signalling and mitochondrial dysfunction	↑ EIF2	Ingenuity Pathway Analysis (IPA)	[137]
				↑ Sirtuin		
BAV-TAA, sTAA	Human	Aortic tissue	Metabolic processes, proteome alterations and immunological processes	↑ LGALS9	Aged *vs* young TAA	[138]
BAV-TAA, sTAA	Human	Aortic tissue from TAV and BAV (non-dilated and dilated ascending aortas)	Inflammatory processes and repair capacity	↑ smooth muscle actin	In TAV and BAV dilated regions *vs* non-dilated	[117]
				↑ α actinin 1		
				↑ α actinin 4		
				↑ myosin light polypeptide 6		
				↑ gelsolin		
AAA, TAA	Human	Non-dilated and dilated aortic tissue	ECM remodelling	↑ Fibrinogen	In dilated *vs* non-dilated TAA	[139]
				↑ Tenascin		
				↑ Thrombospondin-1		
				↑ Decorin		
				↑ Vitronectin		
			Cytoskeletal alterations	↑ Myosin regulatory light polypeptide 9		
				↑ Destrin		
				↑ Filamin-binding LIM protein 1		
				↑ Transgelin		
				↑ Vimentin		
				↑ Myosin light polypeptide 6		
			Proteases inhibition	↑ α-2-macroglobulin		
			Proteolytic processes	↑ MMPs		
sTAA	Human	Aortic tissue	Cytoskeletal alterations, ECM remodelling, morphology, cell-to-cell signalling and interactions	↑ TGF-β signalling mediators	CABG as healthy controls	[140]
BAV-TAA, sTAA	Human	Aortic tissue	TGF-β signalling	↓ LTBP4	BAV *vs* TAV (analysis on released factors)	[142]
BAV-TAA, sTAA, MFS-TAA	Human	Aortic tissue, VSMC and plasma samples	TGF-β signalling	↑ MFAP4	MFS-TAA *vs* non-syndromic TAA	[143]
				↑ PGCA		
				↑ CSPG2		
MFS-TAA	Human	Aortic tissue and VSMC	Mitochondrial dysfunction	↓ PPAR-α ↓ PPAR-γ coactivator 1α ↓ PPAR-δ		[144, 145]
AAA, TAA	Human	Blood	Circulating biomarkers	↑ Kallistatin	Proteomic analyses of postsurgical serum compared with presurgical serum	[146]
				↑ α-2-macroglobulin (only in TAA)		
BAV-TAA	Human	Aortic tissue and plasma	Exocytosis, ECM remodelling, cytoskeleton alterations	↑ RhoA	Disease enrichment analysis, integrated enrichment analysis and canonical pathway enrichment analysis	[147, 148]
				↑ Integrins		
				↑ ADAM10		
				↑ MMP-12		
				↑ TGF-β1		
				↑ GATA4		
				↑ NOTCH3		
Syndromic and non-syndromic TAA	Human	Blood	Circulating biomarkers	↑ ACAN		[149, 150]
MFA-TAA	Human	Blood and iPSC-derived MFS-VSMC	VSMC phenotype switch	↓ Mannose receptor C type 2		[151]
				↓ Transgelin		
				↓ Nestin		
				↑ Integrin		
				↑ MMP2		
				↑ Collagen type 1α		
				↑ Fibronectin 1		

### Metabolomics studies

Metabolomics discipline allows the identification and quantification of metabolite levels, not only in tissue but also in biological fluids (such as blood, urine or cephalorachid fluid) in order to compare different populations and monitor the development of diseases with a non-invasive liquid biopsy technology.

Despite the still limited number of studies, different works have shown nowadays metabolomic alterations in the comparison of TAA samples with controls, suggesting the role of these metabolites as potential predictable biomarkers (Table 5). In particular, the main metabolic differences to date identified concern the pathways of carbohydrate, lipid, amino acid and proteoglycans. However, originally, there were only four metabolomics studies, all conducted on AAA, in which the main metabolic alterations observed in plasma in comparison with healthy control samples were referred to the lipid metabolism, including sphingolipids and lysophospholipids, as well as to the amino acid one (especially the one of the carnitine) [152–155].

**Table 5:** Metabolomic characterisation of aortic diseases

Aneurysm Type	Source		Pathways		Notes	Citations
	Species	Samples	Target	Up/Down		
AAA	Human	Blood	Metabolomic characterisation	↓ Acylcarnities	Fingerprinting analysis for an aneurysm size-based stratification	[152]
				↓ Sphingosine-1-phosphate		
				↓ Sphinganine-1-phosphate		
				↑ Guanidinosuccinic acid		
MFS-TAA, AAA	Human	Aortic tissue and VSMC	Proteomic characterisation	↓ PPAR factors		[144]
AAA	Human	Aortic and AAA thrombus	Inflammation factors	↑ Leukotriene compounds		[153]
				↑ Hippuric acid		
				↑ Lysophosphatidylcholines		
				↑ Hydroxy-oxo-cholanoic acid		
				↑ Pyridoxamine 5’-phosphate		
				↑ 5-oxo proline exchange		
AAA	Human	Plasma	Inflammation and oxidative stress factors	↑ Plasma glucose	Plasma fingerprinting by GC–MS	[154]
				↑ Free phosphate		
				↑ Ketones		
				↑ Fatty acids		
sTAA, BAV-TAA, TAD	Human	Aortic tissue	Metabolites	↑ Sphingomyelin	First metabolomic study on sTAA: targeted FIA-MS/MS metabolomics approach	[156]
Syndromic and non-syndromic TAA	Human–Murine	Plasma	Metabolites	↑ C18-ceramide (TAD)		[158, 159]
				↓ Pyridoxate levels (TAA)		
		Aortic tissue				
Atherosclerotic TAA (aTAA), non-atherosclerotic TAA (naTAA, including MFS samples), atherosclerotic AAA	Human	Aortic tissue	Lipids	↑ Cholesterol (aTAA, naTAA, AAA)		[160]
				↓ Ether-type phosphatidylethanolamines (naTAA)		
				↑ Phospholipids (naTAA)		
				↑ Oxidised cholesterolester (naTAA)		
				↑ Triacylglycerols (naTAA)		
				↑ Prostaglandin D2 (naTAA)		
				↑ 15-LOX metabolites (naTAA)		
LDS-TAA	Human	Aortic tissue	Antioxidant enzymes	↓ Antioxidant enzyme activity		[161]
TAA, TAD	Human–Murine	Aortic tissue from both TAD patients and Fbn1^mgR/mgR^	Metabolites	↑ Proteoglican		[150]
				↑ Versican		
				↑ Perlican		
				↓ Aggrecanases		
				↓ Versicanases		
MFS-TAA	Human	VSMC, aortic tissue and skin samples obtained from MFS patients	TGF-β1, 2 and 3, hyaluronan content, apoptosis, markers of cell migration, and infiltration of vascular progenitor cell	↑ TGF-β1		[163]
				↑ Hyaluronan		

In particular, to classify patients on the basis of aneurysm size (*i.e.*, small or large AAA), Ciborowski et al*.* performed fingerprinting of plasma exploiting a metabolomics strategy. Besides a significant decrease of sphingosine 1-phosphate, sphinganine-1-phosphate and long-chain acylcarnitines (already known as associated with different cardiovascular diseases), the authors also found alterations in the concentration of metabolites of hemoglobin (bilirubin and biliverdin) and of cholesterol (cholanoic acid derivative and bile acid), as well as a plasmatic increase of guanidinosuccinic acid, found as a strong marker of large AAA [152]. Thus, the authors stated that cholesterol, carnitine, and fatty acids may play a key role in the development and progression of AAA. On the contrary, in a counterintuitive manner, although the results showed a strong decrease in sphingolipids, the conclusions of the paper suggest a role of these molecules as potential responsible for aneurysm pathogenesis, by altering the PPAR-γ pathway in VSMC. Interestingly, almost ten years after this study, Verhagen et al*.* confirmed by proteomic analysis these concerns on PPAR factors, which were downregulated in MFS-TAA samples [144].

In another study [153], the authors underlined the importance of the inflammation role in the aortic wall of AAA and intraluminal thrombus, supported by results on increased levels of leukotriene compounds, hippuric acid and lysophosphatidylcholines, hydroxy-oxo-cholanoic acid, and vitamins, such as pyridoxamine 5ʹ-phosphate. Notably, they observed a strong increase of the 5-oxo proline exchange by the aneurysmal wall compared to the healthy aorta, leading to an increase of oxidative stress in the aneurysm. On the contrary, the healthy wall releases more amide fatty acids and vitamin E.

The relationship between inflammation, oxidative stress, metabolic syndrome and AAA was confirmed by Rupérez et al*.* [154], who observed changes in amino acid metabolism and alterations in the carbohydrate and lipids, many of which are associated with diabetes and insulin resistance conditions. In particular, these alterations mainly regarded increased levels of plasma glucose, free phosphate, ketones (acetoacetate, 3-hydroxybutyrate and acetone) and fatty acids.

The fourth study on AAA, performed in 2012 by Pillai and colleagues [155] reported, for the first time, the temporal relationships between local chemical mediators of inflammation and resolution in patients undergoing AAA repair. In details, this study allowed to determine a patient profile, described on the basis of metabolomics results performed on plasma samples: patients may be distinguished between a first profile, fitting with a proinflammatory status throughout the time course, and a second group, who displayed a pro-resolving mediator profile. Since all patients of the study recovered after aortic replacement, these two groups reflected, according to the author suggestions, an early and a delayed resolver population. In order to determine these two profiles, Pillai et al*.* focused on both lipid- and peptide-derived chemical catabolites and their relationship, to better appreciated pro-inflammatory mediators, including vasoactive eicosanoids and the cytokines important to tissue repair [155].

Based on these observations on AAA, in 2017 Doppler et al*.* conducted the first metabolomic study, by using a targeted approach, on tissues of non-syndromic TAA, distinguishing between BAV-associated aneurysms, TAA in patients with TAV and aortic dissection in patients with TAV [156]. Despite no significant differences have been reported between samples of controls, TAV-TAA tissues, BAV-TAA and dissection with TAV, notwithstanding the pathologic specimens, especially in BAV and dissected TAV, showed higher expression levels of sphingomyelin content than controls. This difference is probably due to a reduction/inactivation of sphingomyelinase activity. In fact, although the sphingomyelinase-ceramide pathway is thought to exert pro-inflammatory, pro-oxidative and cell death-inducing activities (resulting in atherosclerosis, aging and cardiovascular events), the findings of this study suggest that classical pro-atherogenic processes may not play a role in these forms of aortic diseases, contrary to what happens in AAA. This study was performed on tissue, and not on serum, firstly to avoid the signal noise of the systemic metabolome and, secondly, to focus only on the metabolic profile of the pathological aortic tissue. However, the results obtained were in agreement with those collected in human serum by Hammad et al*.* [157], suggesting that sphingomyelins SM C16:0 and SM C24:1 could be translated into a serum-based assay.

Because of the targeted approach limitations, in 2022, Yang et al*.*, performed an untargeted metabolomics strategy on plasma samples of syndromic and non-syndromic TAA. The authors found that the alterations in the ceramide metabolism were involved in the development of thoracic aortic dissection, since the levels of these metabolites increased the aortic inflammation through the pathway stimulated by a component of the inflammasome, as NLR family pyrin domain containing 3 (NLRP3) [158]. Distinctly, the authors observed that C18-ceramide content was significantly increased in samples obtained from patients with aortic dissection, but not in TAA, in which, on the contrary, there was a particular decrease of pyridoxate levels, a metabolite of vitamin B6. Interestingly, the association between levels of this compound with AAA has been previously reported, together with its ability to mitigate MFS-TAA murine models, by restoring the canonical TGF-β pathway [159]. Regarding ceramide, by observing the aortic dissection tissues both on human patients and on murine models, an increase in ceramide de novo synthesis pathway has been observed in macrophages. Moreover, the inhibition of this pathway with myriocin significantly reduced β-aminopropionitrile-induced aortic inflammation and dissection in mice. In fact, in vitro studies showed that exogenous administration of C18-ceramide promoted macrophage-driven inflammation and MMP expression through the NLRP3-caspase 1 pathway. On the contrary, the myriocin-mediated inhibition of endogenous ceramide synthesis attenuated lipopolysaccharide-induced macrophage inflammation [158].

Due to the importance of lipid metabolism in different pathophysiological conditions, it is noteworthy to mention a lipidomic study, in which the authors analyzed the lipidic profile in the aortic *tunica media* from normal, border, and aneurysmal tissues, collected from atherosclerotic TAA, non-atherosclerotic TAA (including MFS samples) and atherosclerotic AAA. The comparisons with healthy controls revealed that, while in atherosclerotic TAA and AAA there were lipid alterations in all aortic areas (as visible for the accumulation of cholesterolester), in the non-atherosclerotic TAA this event has not occurred. On the contrary, this form of aneurysm showed decreased phospholipid ether-type PE (also found in atherosclerotic AAA), but grade-associated increases in phospholipids, like phosphatidylcholines and sphingomyelins, cholesterol/cholesterolester, triacylglycerols, and prostaglandin D2 and 15-LOX metabolites. These results suggest a pivotal role of the latter class of molecules in the atherosclerotic aortic aneurysm development and their potential application as markers of transitional risk for the aortic diseases [160].

Concerning the inflammation process, oxidative stress and altered endothelial function, a serum metabolic approach was conducted on MFS patients in 2021 by Bartenbach and colleagues [161]. Here, the authors observed lower levels of histidine, taurine, and PCaeC 42:3 in MFS than in healthy controls, in line with previously mentioned Doppler’s observation on tissue samples of TAV and BAV.

In agreement with this study, the increase of oxidative stress has been observed also in another syndromic non-MFS TAA, such as LDS, in terms of decrease of antioxidant enzyme activity [162].

To better understand differences between various forms of TAA, in 2018 Cikach et al*.* performed a metabolic analysis comparing MFS mouse models and human aortic specimens from patients with TAA and dissection. The results showed an increased production and a dramatic accumulation of proteoglycan and, in particular, aggregates of aggrecan and versican, especially in medial lesions, correlated with increased levels of their mRNA. On the other hand, there was a reduction of aggrecanases and versicanases leading to a reduced proteolytic turnover [150]. According to the author concerns, proteoglycans are key elements for the ECM integrity and homeostasis. However, the accumulation of these ECM compounds could become detrimental not only for the aortic mechanical features, but also in terms of VSMC vitality, by augmenting aortic wall stress levels and defining a deleterious microenvironment. Indeed, it has been observed that the production of versican and hyaluronan is enhanced by TGF-β, together with an increase of intracellular and medial hyaluronan in VSMC and aortic tissue, respectively, in MFS patients [163].

Therefore, considering what has been highlighted above, a metabolomic approach could represent an interesting tool to discover not only new biomarkers, but also new pharmacological targets, by using a non-invasive method. Nevertheless, to date there are only few studies, that still present many limitations (*e.g.*, targeted approach, limited cohorts, observational and cross-sectional studies). Furthermore, as highlighted also for the other -omics, the different forms of TAA have been often considered in pool, and not as distinguished diseases. Although single metabolites to be used as biomarkers to differentiate between disease groups and controls have not been found yet, a combination of several markers may lead to more stable results.

### Integrative multi-omics approaches

Nowadays, the technological advances and the decreased costs of high-throughput experiments have enabled multiple biological layers to be deeply investigated, making possible the exploration of complex biological systems [164]. Consequently, to holistically face complex diseases, it is increasingly important to combine each -omics layer also with clinical data, in order to extrapolate hidden relevant information at several levels [165]. Indeed, studying disease from the genome to phenome layer, integrative multi-omics approaches have the potential to provide researchers with a greater understanding of the flow of information, from the original cause of disease to the functional consequences or relevant interactions, and to elucidate novel pathophysiological insights [166].

However, although integrative approaches are powerful strategy to decipher the mechanistic details, the complexity of related data analysis grows in proportion to the size of available data. Indeed, multi-omics investigations usually relies on the building of complex interactome networks and the development of accurate prediction models for disease diagnosis, exploiting graphs theory and machine learning approaches, respectively. Both the analyses come with hard challenges [167, 168]. In the first case, it is not easy to infer causality between interactions, as most of links are estimated by simple correlations or co-expressions; in addition, to explore the interactions between thousands or millions of entities (*e.g.*, genes, proteins, metabolites) a huge amount of computational resources are needed. Regarding machine learning analysis, the harmonization of data and the ‘course of dimensionality’ pose crucial issues; the first problem occurs when trying to combine databases which have both different observations and features; on the other hand, the second question occurs when we build classification models with a huge number of features in compare with the number of samples.

**Fig. 3:**
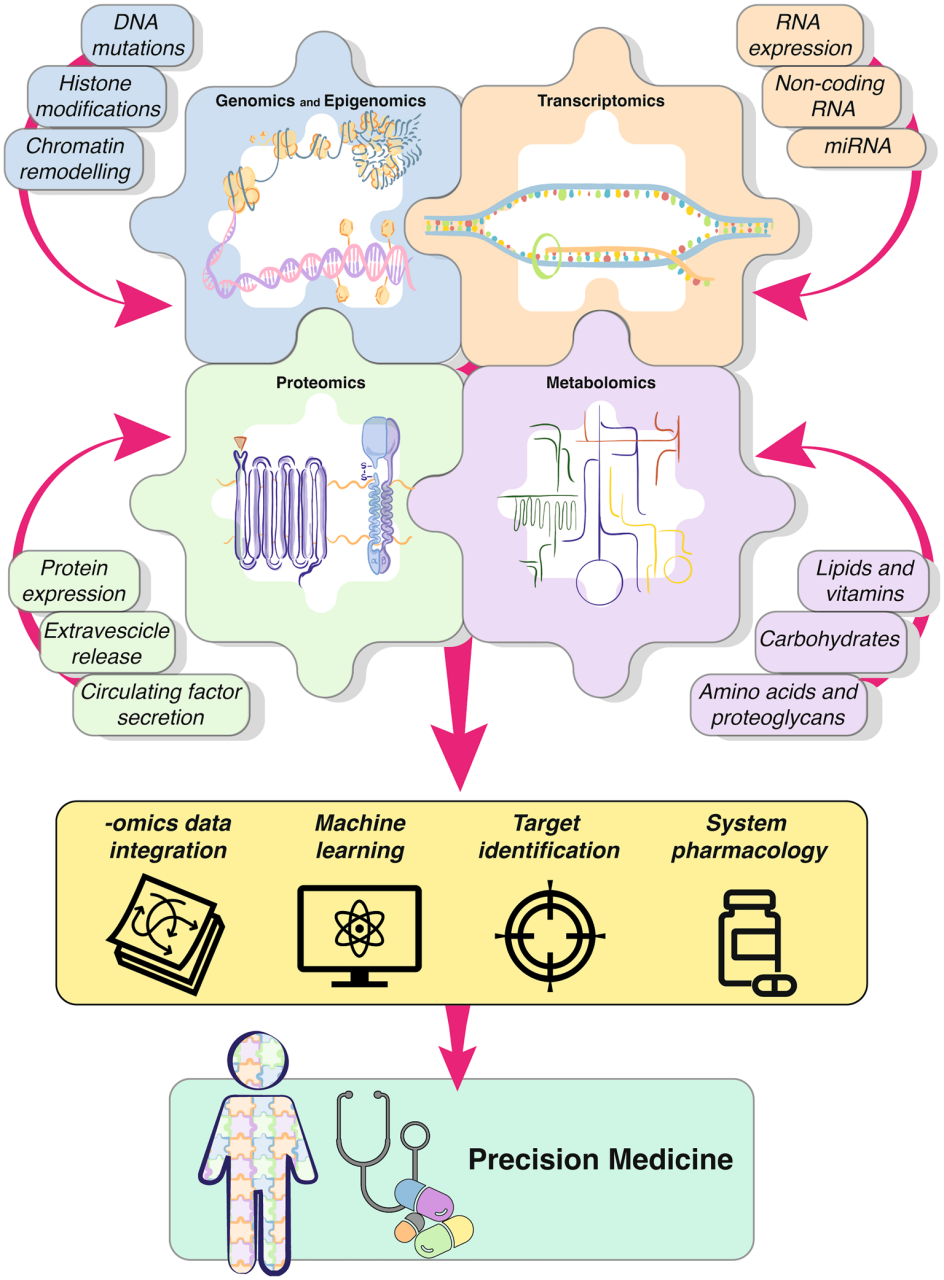
**–** Multi-omics tool to solve the puzzle of precision medicine in thoracic aortic disease. Nucleic acids, proteins, extracellular vesicles and metabolites derived from biological specimens or cell culture models, as previously depicted in Fig. 2, are starting points to generate multiple high-throughput next-generation data, composing a “puzzle” of interrelated information. “–Omics” read-out integration and machine learning approaches like bioinformatics are then performed to evaluate differential expression/enrichment of specific molecules between physiological and pathological conditions. Likewise, this strategy allows to reveal unknown altered mechanisms/patterns, thus enabling the identification of potential predictive/prognostic biomarkers and pharmacological targets. The subsequent application of system pharmacology study provides a more targeted and precise medicine, tailored to the individual patient with thoracic aortic aneurysm

Overall, as integrative multi-omics approaches are powerful tool to arise new relevant information from tangled molecular scenarios, new studies combining multiple -omics layers on TAA are strongly desired to identify key players on its pathogenesis and to propose effective therapeutic strategies or drugs (Fig. 3).

## Conclusions

The objectives of this paper are mainly two: the first is to provide a comprehensive vision on the multitude of data collected nowadays by different -omics techniques (epigenomics, transcriptomics, proteomics, and metabolomics) in dissecting the TAA issue. More importantly, the second aim of this work has been achieved only if the readers have grasped the difficulty of finding a unique approach to study and solve the complex picture regarding molecular mechanisms involved in the formation and progression of the different types of TAA. Indeed, merging the “forest” of -omics data here reviewed, we can assume that alterations in mediators of TAA pathways frequently occur in TGF-β signaling (*e.g.*, *ACTA2, SMAD2*) and in the subsequent pro-fibrotic events (*e.g.*, CTGF, MMPs), or in proteins involved in cell/extracellular structures (*e.g.*, contractile/ECM proteins). Furthermore, the analyses performed highlighted an important role played also by other processes, as inflammation, oxidative stress, mitochondrial dysfunction (in terms of mitochondrial respiration) and the glycolysis/gluconeogenesis pathways. At last, novel mediators, recently observed by metabolomics studies (*e.g.*, carbohydrate, lipid, amino acid, proteoglycans), have been added to the list of molecules and pathway specifically involved in TAA, highlighting unprecedented marks, potential useful for diagnosis and therapy of TAA.

In conclusion of this review, it is important to highlight fundamental issues. At first, a unique protocol to obtain different preparations at once for all the -omics techniques from one single sample is not yet completely determined. Indeed, protocols for epigenomics/genomics/transcriptomics analyses often involve cell lysis steps, aimed to well isolate nuclei and RNA [169], but also leading to the loss of biological material, which can be considered valuable for other -omics applications (*e.g.*, proteomics and metabolomics). On the other hand, protocols adopted for proteomics preparations most always involve TAA tissue pulverization at extremely low temperature and the subsequent addition of urea-based buffers, supplemented with ammonium bicarbonate and surfactants/detergents. All these steps unfortunately may lead to the nucleic acid loss.

Secondly, always in regard of standardization issue, the choice of correct ‘controls’ for the adequate comparisons may be considered of higher importance. Noteworthy, this review has frequently pointed out the attention on this issue. All these concerns lead to the necessity of more comprehensive studies, in which the evaluation of -omics features in TAA will be performed on well-defined specimens, in order to better dissect, for example, the differences between syndromic and sporadic TAA. Altogether, heterogeneity of sample extraction protocols as well as data collection strongly contributes to a significant gap between clinical and basic research, limiting the translation of -omics approaches in clinical practice [170].

The multi-layer analysis of the -omics, together with the adoption of sophisticated bioinformatic tools, should be implemented, at the end, with system pharmacology approaches by using specific drug prediction databases, allowing drug repurposing. This complex approach could lead to striking results in terms of translation from in vitro*/*in vivo studies on TAA patients. An elegant example is the already mentioned study of Hansen et al*.* of 2019, in which the authors integrate the transcriptomics results (obtained from human and mice samples) with a computational approach. In this paper, the authors were able to identify a novel pharmacological approach to limit MFS-TAA progression, based on an available drug [130].

In conclusion, as far as TAA concerned, despite progress in the knowledge of its pathogenetic basis, the determination of precise clinical markers of pathology and disease progression is still lacking. In this context, -omics technologies, and overall their integration, could represent a useful strategy to discover relevant pathophysiological factors that can be used as predictive or prognostic disease markers [171], as well as potential therapeutic target to better distinguish and treat the different forms of TAA.

## Supplementary Information

**Additional file 1: Table S1.** Animal models for thoracic aortic aneurysms.

## Data Availability

Not applicable.
