# Supplementary material for: Multi-omics in thoracic aortic aneurysm: the complex road to the simplification
Source: Cell Biosci. 2023 Jul 20;13:131. doi: 10.1186/s13578-023-01080-w (PMC10357728; doi:10.1186/s13578-023-01080-w)
Supplement: Supplementary file 1 — Additional file 1: Table S1. Animal models for thoracic aortic aneurysms. [file 13578_2023_1080_MOESM1_ESM.docx]

Multi-omics in thoracic aortic aneurysm:

the complex road to the simplification

Sara Rega, MSc^1,2^, Floriana Farina, PhD^3,4^, Silvia Bouhuis, BS^1^, Silvia de Donato, MD^1^,

Mattia Chiesa, PhD^5,6^, Paolo Poggio, PhD^2^, Laura Cavallotti, MD, PhD^7^, Giorgia Bonalumi, MD^7^,

Ilaria Giambuzzi, MD^7,8^, Giulio Pompilio, MD, PhD^1,7,9^, Gianluca L. Perrucci, PhD^1,^*

^1^ Unit of Vascular Biology and Regenerative Medicine, Centro Cardiologico Monzino IRCCS, Milan, Italy

^2^ Unit for the Study of Aortic, Valvular and Coronary Pathologies, Centro Cardiologico Monzino IRCCS, Milan, Italy

^3^ Institute for Cardiovascular Prevention (IPEK), Ludwig-Maximillians-Universität (LMU) München, D-80336 Munich, Germany

^4^ German Center for Cardiovascular Research (DZHK), Partner Site Munich Heart Alliance, D-80336 Munich, Germany

^5^ Bioinformatics and Artificial Intelligence facility, Centro Cardiologico Monzino IRCCS, Milan, Italy

^6^ Department of Electronics, Information and Biomedical engineering, Politecnico di Milano, Milan, Italy

^7^ Department of Cardiovascular Surgery, Centro Cardiologico Monzino IRCCS, Milan, Italy

^8^ Department of Clinical Sciences and Community Health, Università degli Studi di Milano, Milan, Italy

^9^ Department of Biomedical, Surgical and Dental Sciences, Università degli Studi di Milano, Milan, Italy

**Short title:** Multi-omics in thoracic aortic aneurysm

***** **Corresponding author**

Gianluca L. Perrucci, PhD

Unit of Vascular Biology and Regenerative Medicine

Centro Cardiologico Monzino IRCCS, Milan, Italy

Tel.: +39 02 58002754

email: gianluca.perrucci@cardiologicomonzino.it

**Keywords:** Thoracic aortic aneurysm; Epigenomics; Transcriptomics; Proteomics; Metabolomics.

Additional files

**Immunohistochemistry**

Aortic tissue samples were collected as previously described [56]. Briefly, dilated region of thoracic aortas (TAA) from patients undergone aortic surgical substitution were collected in the surgery room. The healthy control samples were provided by Unit of Pathology of “ASST Santi Paolo e Carlo” of Milan. All patients gave written informed consent for tissue collection and the procedure was approved by ASST FBF-Sacco Ethics Committee (Prot. N. 39138/2016, Milan, Italy). Aortic samples were stored in serum-free Medium 231 (Life Technologies, Carlsbad, CA, USA) and then divided into fragments dedicated to histology or aortic cell isolation. Aortic fragments for histological analyses were fixed in Carnoy’s solution, embedded in paraffin and cut to obtain 5-µm-thick sections from each sample. To evaluate collagen I expression pattern in aortic tissue, an immunohistological assay was performed. Briefly, sections were kept 20 minutes at 99 °C in Tris/EDTA pH 9.0. Endogenous peroxidase activity was quenched with 3% H_2_O_2_ for 10 minutes, then sections were incubated with primary antibody against COL1A1 (Cell Signaling Technology, Danvers, MA, USA) over-night (O/N) at 4° C. After the incubation of sections with secondary antibody, the staining was performed with 3,3-diaminobenzidine (DAB) as a chromogen. Slides were immunostained in the same batch, including negative controls. As negative controls, incubation with species- and isotype-matched IgGs was performed in place of primary antibodies. Slides were viewed with AxioImager microscope equipped with AxioCam camera (Carl Zeiss, Oberkochen, Germany).

**Vascular smooth muscle cell isolation**

As previously described [56], aortic samples for cell isolation procedure were stored in serum-free Medium 231 (Thermo Fisher Scientific, Waltham, MA, USA), then washed with Phosphate Buffer Saline (PBS), minced and digested O/N at 37° C in a solution of 2 mg/mL collagenase type II (Worthington Biochemical Corporation, Lakewood, NJ, USA) in complete Medium 231, supplemented with Smooth Muscle Growth Supplement (SMGS, Thermo Fisher Scientific, Waltham, MA, USA). The result of tissue digestion was filtered with 100 µm cell strainer, pelleted and plated in complete Medium 231. To improve vascular smooth muscle cell (VSMC) growth, the day after isolation, the medium was changed to remove the residual erythrocytes. As control, commercially available VSMC were used (Lonza, Basel, Switzerland).

**Immunofluorescence**

Control VSMC (HC-VSMC) and VSMC obtained from TAA patients were plated on histologic round glass on the bottom of a 24-multiwell plate and placed in growth for 24 hours with 95% humidity and 5% CO_2_. Glasses were rinsed with PBS solution and soaked for about 15 minutes in a solution of 4% paraformaldehyde (PFA). The phalloidin, conjugated to AlexaFluor-488 (Thermo Fisher Scientific, Waltham, MA, USA), was incubated 1 hour at room temperature. Nuclei were stained with Höchst (Thermo Fisher Scientific, Waltham, MA, USA). Slides were viewed with ApoTome2 microscope equipped with AxioCam camera (Carl Zeiss, Oberkochen, Germany).

Additional table

**Table S1 – Animal models for thoracic aortic aneurysms.**

| **Model** | | **Type of model** | | **Notes** | **Ref.** |
| --- | --- | --- | --- | --- | --- |
| **Species** | **Name** | **Molecular target** | **Method** |  |  |
| Murine (*Mus musculus*) | Fbn1^C1039G/+^ | *Fibrillin1* | Site-directed mutagenesis. | Heterozygous mice develop proximal aortic aneurysms. A similar mutation in human (Cys1039Tyr) is known to cause classic manifestations of Marfan (MFS) syndrome. Heterozygous mice develop also mitral valve thickenings, pulmonary alveolar septation defects, mild thoracic kyphosis, and skeletal myopathy. | [1] |
| Murine (*Mus musculus*) | Fbn1^mgR/mgR^ | *Fibrillin1* | A targeting vector containing a PGKneo cassette was electroporated into embryonic stem cells. An unequal crossover resulted in no loss of endogenous sequence for this "mgR" mutant allele. | Homozygotes develop medial calcification, the inflammatory-fibroproliferative response, and inflammation-mediated elastolysis with MFS-like manifestations. | [2] |
| Murine (*Mus musculus*) | Fbln4^E57K/E57K^ | *Fibulin4* | A neomycin (neo) resistance cassette was inserted in reverse orientation downstream of exon 4 of the epidermal growth factor-containing fibulin-like extracellular matrix protein 2 gene. A point mutation was introduced in exon 4, resulting in a missense mutation, E57K, found in patients with autosomal recessive *cutis laxa* (ARCL) 1B. | Fbln4^E57K/E57K^ mice display aortic aneurysm, as well as abnormalities in multiple organ systems, including loose skin, bent forelimb, tortuous artery and pulmonary emphysema. ARCL 1B is characterized also in human by loose skin, arterial tortuosity, lung emphysema and skeletal abnormalities. | [3] |
| Murine (*Mus musculus*) | Fbln4^SMKO^ | *Fibulin4* | Heterozygous *Fbln4^+/-^* were bred with SM22-Cre mice, as reported by Huang *et al.* in 2010. | The vascular phenotype in Fbln4 mutant mice is remarkably similar to a subset of human thoracic aortic aneurysms caused by mutations in SMC contractile genes. | [4] |
| Murine (*Rattus norvegicus*) | JCR:LA-cp | *cp* | The JCR:LA-cp rat is one of the outbred strain of "corpulent" rat (*i.e.*, leading a homozygous mutation of the autosomal recessive gene *cp*). | These rats show cystic medial necrosis in TAA. Animals homozygous for the gene (*cp/cp*) become obese, insulin resistant, and hypertriglyceridemic. JCR:LA-cp rat develops a vasculopathy with atherosclerotic lesions and associated ischemic myocardial lesions. The cardiovascular disease of this model is strongly correlated with the hyperinsulinemia. | [5] |
| Murine (*Mus musculus*) | AngII-infused ApoE^-/-^ | Renin-angiotensin system | AngII is delivered by implanted osmotic pump. Part of both exon and intron 3 of the *Apoe* was replaced with a neomycin resistance (neo) cassette. | This model is frequently used to study aneurysmal lesions, but these mice may develop both TAA and AAA. | [6] |
| Murine (*Mus musculus*) | BAPN administration + AngII infusion | *LOX/LOXL* activity (under investigation) | BAPN is administered by drinking water, less frequently by diet; AngII is delivered by implanted osmotic pump. | This model develops both TAA and AAA. | [7, 8] |
| Murine (*Mus musculus*) | TAC | // | The aortic constriction is placed by tying suture wire with a small needle, between the right innominate and left carotid artery. After two knots, the needle is promptly removed leading to an aortic constriction of approximately 60-80% of original diameter. | Ascending thoracic aortic remodelling after TAC is characterized by medial thickening, adventitial hyperplasia and collagen deposition, beside left ventricular hypertrophy. | [9] |
| Murine (*Mus musculus*) | // | // | The distal half of descending thoracic aorta was wrapped with a sponge soaked in CaCl_2_ for 15 minutes. | Previous studies focusing on the infrarenal abdominal aorta have shown successful induction of aneurysms at both 3 and 10 weeks of age. | [10] |
| Murine (*Rattus norvegicus*) | // | // | The descending aorta of animals (5^th^ intercostal space) was dissociated from left subclavian artery for 1cm. Cotton yarn with saline or porcine pancreatic elastase was wrapped around the exposed aorta for 20 minutes. | In this model of descending TAA, the phenotypic switch of VSMC has been observed. | [11] |
| Swine  (*Sus scrofa*) | // | // | Aortic media and intima were resected. After intima excision, only the adventitial layer was sutured and closed. | The saccular aneurysm is localized between distal arch and descending aorta (left 4–5^th^ intercostal space). The disease model animals do not fully reflect human clinical pathology. | [12] |
| Swine  (*Sus domesticus*) | // | // | Intra-adventitial injections of collagenase and peri-adventitial application of crystalline CaCl_2_. | This protocol induced a dilatation of the descending thoracic aorta, reproducible and consistent with that of aneurysm formation. | [13] |
| Canine  (*Canis familiaris*) | // | // | The proximal descending thoracic aorta (3^th^–4^th^ intercostal space) of Mongrel dog was isolated and incised. An oval patch of pericardium was resected and sewn onto a longitudinal aortotomy with the visceral surface of the patch on the aortic flow side. | The surgical procedure determined a saccular aneurysm (proximal descending TAA). | [14] |
| Canine  (*Canis familiaris*) | // | // | The Beagle dog descending thoracic aorta (7^th^ intercostal space) was mobilized and incised. A vein patch with the left external jugular vein was isolated and sewn on aortotomy. | This model of drug-eluting stent-grafts as thoracic descending aneurysm treatment may improve results of the endovascular therapeutic approaches. | [15] |
| Canine  (*Canis familiaris*) | // | // | Thoracic aorta was isolated and dissected in Mongrel dogs. A nitinol-polyester self-expandable endograft was sewn onto the left anterolateral side of the aorta. | One of the study limitations is the different size of canine thoracic aorta and the provided endoprosthesis. | [16] |

Additional bibliography

1. Judge DP, Biery NJ, Keene DR, Geubtner J, Myers L, Huso DL, Sakai LY, Dietz HC: **Evidence for a critical contribution of haploinsufficiency in the complex pathogenesis of Marfan syndrome**. *The Journal of clinical investigation* 2004, **114**(2):172-181.

2. Pereira L, Lee SY, Gayraud B, Andrikopoulos K, Shapiro SD, Bunton T, Biery NJ, Dietz HC, Sakai LY, Ramirez F: **Pathogenetic sequence for aneurysm revealed in mice underexpressing fibrillin-1**. *Proceedings of the National Academy of Sciences of the United States of America* 1999, **96**(7):3819-3823.

3. Igoucheva O, Alexeev V, Halabi CM, Adams SM, Stoilov I, Sasaki T, Arita M, Donahue A, Mecham RP, Birk DE *et al*: **Fibulin-4 E57K Knock-in Mice Recapitulate Cutaneous, Vascular and Skeletal Defects of Recessive Cutis Laxa 1B with both Elastic Fiber and Collagen Fibril Abnormalities**. *The Journal of biological chemistry* 2015, **290**(35):21443-21459.

4. Huang J, Davis EC, Chapman SL, Budatha M, Marmorstein LY, Word RA, Yanagisawa H: **Fibulin-4 deficiency results in ascending aortic aneurysms: a potential link between abnormal smooth muscle cell phenotype and aneurysm progression**. *Circulation research* 2010, **106**(3):583-592.

5. Pung YF, Chilian WM, Bennett MR, Figg N, Kamarulzaman MH: **The JCR:LA-cp rat: a novel rodent model of cystic medial necrosis**. *American journal of physiology Heart and circulatory physiology* 2017, **312**(3):H541-H545.

6. Daugherty A, Manning MW, Cassis LA: **Angiotensin II promotes atherosclerotic lesions and aneurysms in apolipoprotein E-deficient mice**. *The Journal of clinical investigation* 2000, **105**(11):1605-1612.

7. Sawada H, Beckner ZA, Ito S, Daugherty A, Lu HS: **beta-Aminopropionitrile-induced aortic aneurysm and dissection in mice**. *JVS Vasc Sci* 2022, **3**:64-72.

8. Fashandi AZ, Hawkins RB, Salmon MD, Spinosa MD, Montgomery WG, Cullen JM, Lu G, Su G, Ailawadi G, Upchurch GR, Jr.: **A novel reproducible model of aortic aneurysm rupture**. *Surgery* 2018, **163**(2):397-403.

9. Kuang SQ, Geng L, Prakash SK, Cao JM, Guo S, Villamizar C, Kwartler CS, Peters AM, Brasier AR, Milewicz DM: **Aortic remodeling after transverse aortic constriction in mice is attenuated with AT1 receptor blockade**. *Arteriosclerosis, thrombosis, and vascular biology* 2013, **33**(9):2172-2179.

10. Ikonomidis JS, Gibson WC, Gardner J, Sweterlitsch S, Thompson RP, Mukherjee R, Spinale FG: **A murine model of thoracic aortic aneurysms**. *The Journal of surgical research* 2003, **115**(1):157-163.

11. Mao N, Gu T, Shi E, Zhang G, Yu L, Wang C: **Phenotypic switching of vascular smooth muscle cells in animal model of rat thoracic aortic aneurysm**. *Interact Cardiovasc Thorac Surg* 2015, **21**(1):62-70.

12. Fukushima S, Ohki T, Koizumi M, Ohta H, TakahasHi T, Okano HJ: **A reproducible swine model of a surgically created saccular thoracic aortic aneurysm**. *Exp Anim* 2021, **70**(2):257-263.

13. Eckhouse SR, Logdon CB, Oelsen JM, Patel RK, Rice AD, Stroud RE, Wince WB, Mukherjee R, Spinale FG, Ikonomidis JS *et al*: **Reproducible porcine model of thoracic aortic aneurysm**. *Circulation* 2013, **128**(11 Suppl 1):S186-193.

14. Li W, Xu K, Ni Y, Zhong H, Bi Y: **A canine model of proximal descending thoracic aortic aneurysm created with an autologous pericardial patch**. *Ann Thorac Cardiovasc Surg* 2013, **19**(2):131-135.

15. Kajimoto M, Shimono T, Hirano K, Miyake Y, Kato N, Imanaka-Yoshida K, Shimpo H, Miyamoto K: **Basic fibroblast growth factor slow release stent graft for endovascular aortic aneurysm repair: a canine model experiment**. *J Vasc Surg* 2008, **48**(5):1306-1314.

16. Formichi M, Marois Y, Roby P, Marinov G, Stroman P, King MW, Douville Y, Guidoin R: **Endovascular repair of thoracic aortic aneurysm in dogs: evaluation of a nitinol-polyester self-expanding stent-graft**. *J Endovasc Ther* 2000, **7**(1):47-67.
